# Influence of aerobic exercise training on mice gut microbiota in Parkinson’s disease

**DOI:** 10.55730/1300-0152.2617

**Published:** 2022-04-25

**Authors:** Tianlun FAN, Xiating LI, Xiang ZHANG, Jing ZHANG, Lichun SUN, Jingjing CHEN, Chuan FU

**Affiliations:** 1 Department of Rehabilitation Medicine, the First Affiliated Hospital of Hainan Medical College, Hainan, China; 2 Department of Neurology, the First Affiliated Hospital of Hainan Medical College, Hainan, China; 3 Department of Ultrasound Medicine, the First Affiliated Hospital of Hainan Medical College, Hainan, China

**Keywords:** Parkinson’s disease, aerobic exercise training, gut microbiota, 16S rRNA gene sequencing

## Abstract

Accumulating evidence shows that gut microbial dysbiosis may represent a risk factor for Parkinson’s disease (PD). Exercise has a positive effect on microbiota in general. The effect of aerobic exercise training (AET) on the gut microbial environment in PD remains to be explored. Here, we performed the 16S rRNA gene sequencing on feces from sham operated-mice (sham), PD mice model, and mice receiving AET (AET). Results indicated that AET had no remarkable effect on species richness and bacterial diversity of PD mice. The relative abundance of the *Bacteroidetes* was reduced, while *Firmicutes*, *Actinobacteria*, *Lactobacillaceae*, *Streptococcaceae*, *Lactobacillus*, *Streptococcus*, *Lactococcus*, *Lysinibacillus*, *Pelomonas*, and *Prevotellaceae_UCG–001* were increased in PD mice compared with those of sham operated-mice, whereas AET partly rescued their abundance. Additionally, the composition proportion of beneficial *Lactobacillus_gasseri* and u*ncultured_Erysipelotrichales_bacterium* significantly increased in AET mice compared to PD mice. Moreover, discriminative bacteria, such as *Bacilli*, *Lactobacillales*, *Lactobacillaceae*, *Lactobacillus*, and *Lactococcus* were identified as a specific taxon in AET mice. Here we provide evidence that AET can improve the gut microbiota of PD mice.

## 1. Introduction

Parkinson’s disease (PD) is a multi-factorial neurodegenerative disease mainly characterized by motor and nonmotor features (Van Den Eeden et al., 2003). Numerous motor symptoms include bradykinesia, resting tremors, rigidity, and late postural instability (Kalia and Lang, 2015). While the nonmotor signs include olfactory, gastrointestinal (GI), cardiovascular, and urogenital systems disorders (Cersosimo et al., 2013). In brief, PD has a negative impact on patients and their families both in mental impact and economic pressure (Chong et al., 2012). It is thus urgent to find a new sally port.

Recently, there is a realization that the development of PD is influenced by the brain-gut axis (Mayer et al., 2015). For instance, people with PD always manifest in gut microbiome dysbiosis and GI inflammation (Keshavarzian et al., 2015; Hill-Burns et al., 2017). Extensive research revealed that the gut microbiota alterations are implicated in PD pathogenesis (Pfeiffer, 2013). It was reported that the relative abundance of gut microbiota was associated with postural and gait of PD patient (Scheperjans et al., 2015). In addition, several studies exert similar trends in the alteration of microbial composition in individuals with PD, and a decrease in commensal bacteria (e.g., *Firmicutes*) and an increase of pathogenic gram-negative bacteria (*Escherichia*, *Enterobacteriaceae*, *Proteobacteria sp*.) and mucin-degrading *Verrucomicrobiaceae* (Unger et al., 2016; Li et al., 2017).

Although studies on pharmacological therapies for PD have obtained some success, the benefits are often negligible and unsustainable (Szeto and Lewis, 2016). Accumulating evidences suggest that high intensity exercise can be part of the medical management of PD. For example, several evidence demonstrates that aerobic exercise has a protective role in memory and executive dysfunction, and ameliorate severity of depression in otherwise healthy older adults (Blumenthal et al., 1999; Erickson et al., 2011). Animal studies also suggest that exercise can result in an increase levels of antioxidant markers and thus alleviate neuroinflammation that is a main pathological feature of PD (Svensson et al., 2015). Additionally, the positive influence of exercise on microbiota has been widely studied, including enhancing colon health, augmenting the diversity of microbiota and the balance between pathogenic and beneficial bacterial communities (J. M. Evans et al., 2013; Allen et al., 2015). However, the effect of aerobic exercise on altering the composition of the gut microbiota in PD has not yet been studied.

PD mice models have been established in parallel to undergo aerobic exercise training (AET) for PD mice in our previous study (Zhang et al., 2020). In this study, the 16S rRNA sequencing was performed for the intestinal contents from sham mice, PD mice, and PD mice receiving AET to evaluate whether aerobic exercise restore the gut microbiome disorder in PD mice.

## 2. Materials and methods

### 2.1. Animals

According to our previous study (Zhang et al., 2020), the experiments were performed on 15 male C57BL/6 J mice of 10–12 weeks. The animals were assigned to three groups of five mice each at random: sham operated-mice (sham) group, PD group and aerobic exercise training (AET) group. Animals were kept in an incubator of 12 h light-dark cycle with constant temperature (20–24 °C) and humidity and provided ad libitum access to food and water throughout the research. All animals were acclimatized for 1 week prior to the experiment starting. The sham group mice were injected intraperitoneally with equivalent volumes of normal saline for 7 days. The mice were administered intraperitoneally with 1-methyl-4-phenyl-1, 2, 3, 6-tetrahydropyridine (MPTP) at 30 mg/kg for 7 days to construct PD mouse models. The sham and PD groups did not receive any training. AET group began training after the first MPTP injection, they received grasping, rotating, walking and balance training with a circular mesh instrument of 100 cm in length and 60 cm in diameter. Moreover, a square wooden bar 170 cm long and 2 cm wide in diameter was placed 7 cm away from the ground as a balance beam for mice to walk on, mainly to enhance the balance ability of AET group. The frequency of both exercises is once a day, 6 days a week, 30 min each time, a total of 4 weeks. All experimental procedures adhered to compliance on the basis of Animal Ethics Committee of Hainan Medical College.

### 2.2. Behavioral assessment

Behavioral assessment of mice after 0, 2, and 4 weeks of training was performed by swimming and pole tests.

In the swimming tests, the tested mice were put into a 20*30*20 cm size water tank with a water temperature of 22–25 °C. The maximum evaluation time is 1 min. The scoring criteria are as follows: 3 points will be scored if the mouse can swim continuously; if the mouse only floated and swam occasionally, 2 points were scored; mice who floated on one side of their body and swam only occasionally with their hind limbs scored 1 point. If only the mouse’s head floated but its hind legs sank, it was scored 0 points. Finally, calculate the total score of each group.

The pole test process was as follows: A foam ball with a diameter of 2.5 cm was fixed to a wooden pole with a length of 50 cm and a diameter of 1 cm. Gauze was wrapped on the top of the pole and the ball to prevent slipping. The mice were placed upside down on the ball, and the time required for the mice to complete the following 3 movements was recorded. Including the time for the mouse to climb down from the small ball to complete the full length of the wooden pole, and the time of mice climb the upper or lower part of the wooden pole. Then according to the following standard score: three points for completing any part of the action within 3 s, two points in less than six s. One point is scored for more than 6 s or for falling off the rod. The average is taken as the final score of the experiment. If the mice stops halfway or reverse crawling, it will not be recorded and retested.

### 2.3. HE staning

Brain tissue samples were collected after 0, 2, and 4 weeks of training for HE staining. Samples were fixed with 10% formaldehyde solution and embedded in paraffin. Subsequently, brain tissue was cut 4~5 μm thick and stained with hematoxylin solution for 5 min, destained with 1% hydrochloric acid alcohol. Following the sections were dehydrated with ethanol, rinsed with xylene, mounted with neutral gum and observed under an optical microscope (Olympus, × 40).

### 2.4. Library preparation and sequencing

The fecal samples collected after 4 weeks training. The fecal samples of each mouse were collected freshly with fecal collection container, immediately frozen and stored at −80 °C. Total DNA of each feces sample was isolated using QIAamp 96 PowerFecal QIAcube HT Kit (Qiagen). DNA integrity was monitored by agarose gel electrophoresis. PCR was performed to amplify the 16S rDNA V3-V4 region using universal bacterial primers 343F (5′-TACGGRAGGCAGCAG-3′) and 798R (5′-AGGGTATCTAATCCT-3′). Two rounds PCR were performed for establishing library. The products of PCR were purified with AMPure XP magnetic beads (Beckman). The second-round PCR purified products were detected using agarose gel electrophoresis and NanoDrop 2000 spectrophotometer (Invitrogen), quantified using the Qubit dsDNA Assay Kit (Life Technologies). Subsequently, The V3-V4 16S rDNA libraries were sequenced on an Illumina MiSeq platform (Illumina).

### 2.5. Quality control of raw sequencing data

The paired-end raw reads were filtered using Trimmomatic (version 0.35) and checked for average base quality using the moving window method (Bolger et al., 2014). Subsequently, the paired-end filtered reads were merged using Flash (version 1.2.11) based on overlapped reads of at least 10 bp and at most 200 bp at the paired-end read. The ambiguous bases, sequences with high repeat regions of single bases and sequences with too short lengths were removed using Split_libraries (version1.8.0) to obtain clean tags, and retaining sequences whose base quality score Q20 reached at least 75%. Meanwhile, chimera sequences in clean tags were detected and removed using UCHIME (version2.4.2) to obtain valid tags for operational taxonomic units (OTUs) division.

### 2.6. Bioinformatics analysis

OTUs clustering were carried at a 97% similarity threshold using Vsearch software. The representative sequences of each OTU were picked out using QIIME and all representative sequences were annotated using RDP classifier algorithm according to Greengenes database (Confidence interval > 0.7). Subsequent analysis of community structure distribution, alpha diversity, beta diversity analysis was all executed according to these output normalized data. Linear discriminant analysis (LDA) coupled with effect size measurements (LEfSe) based on Phylogenetic Investigation of Communities by Reconstruction of Unobserved States (PICRUSt) was utilized to investigate how classification differences among intestinal microbiota of three groups influence the contribution of different species to difference. LEfSe analysis was carried out under the following conditions: the p value for the factorial Kruskal–Wallis test among classes was 0.05 and the threshold on the logarithmic LDA score for discriminative features was 3.0. Relative abundances in each group were compared using the one-way ANOVA (LSD) via software SPSS 20. The p-value less than 0.05 was considered to be statistically significant.

## 3. Results

### 3.1. Behavior assess

**Figure 1:**
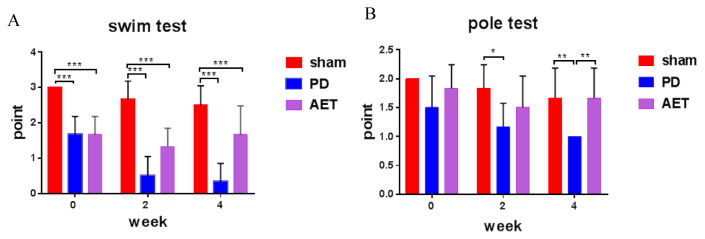
Behavior assess of aerobic exercise training (AET) of mice with PD. **(A)** A swim test was conducted in mice at weeks 0, 2, and 4 after injection. **(B)** A pole test was conducted in mice at weeks 0, 2, and 4 after injection. The Y-axis represents the average score of mice in swim and pole tests. *Represents p < 0.05, **represents p < 0.01, ***represents p < 0.001.

To test this PD mice model, behavioral assessment of mice after 0, 2, and 4 weeks of training after injection was performed by swimming and pole tests. In the swimming test, the scores of sham group were significantly higher than those of AET and PD mice. After 4 weeks of training, the swimming ability of AET group was significantly improved compared with PD group (Figure 1A). Also, in the pole test, the performance of mice in the AET group was significantly improved after 4 weeks of training compared with the PD group (Figure 1B). The rod-climbing experiment and swim test in this paper have proved the successful establishment of PD mouse model.

### 3.2. HE staining

**Figure 2:**
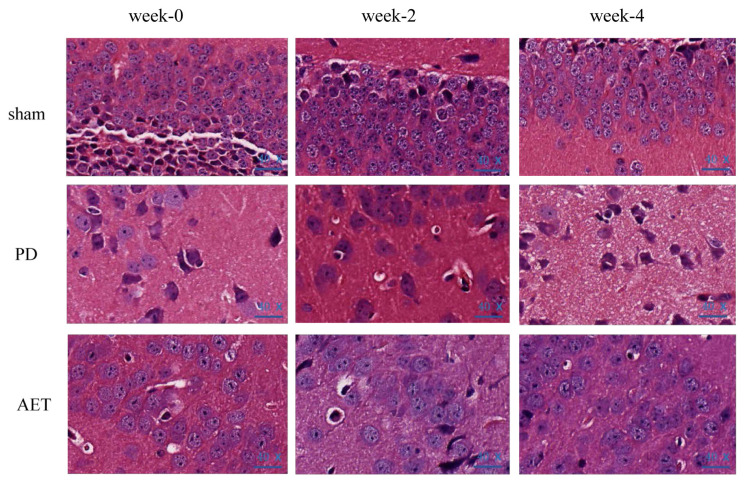
Hematoxylin-eosin staining of mice brain tissues in the sham, PD, and AET group (40 ×).

To further explore the effects of AET on the brain morphology of mice, HE staining was used to observe the pathological differences of brain tissues in each group. As shown in Figure 2, the substantia nigra pars compact (SNpc) of sham group showed scattered, large multipolar neurons with surrounding neutrophils and no pathological changes. The morphology of SNpc cells in PD group changed and the number of SNpc cells decreased. The cytoplasm and nucleus of SNpc cells were stained deeply. Compared with PD group, the changes of SNpc cells in AET group were less, and most SNpc cells had normal structure. In addition, SNpc demonstrates scattered large multipolar cells and surrounding neutrophils. It indicated that AET improves the pathological features of PD mice.

### 3.3. Effect of AET on bacterial diversity of gut microbiota associated with PD

**Figure 3:**
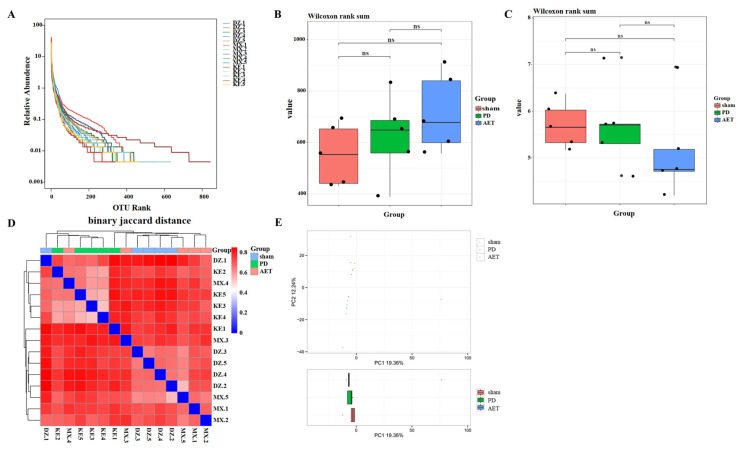
Feature of fecal microbiota in sham-operated mice, Parkinson’s disease (PD) mice, mice receiving aerobic exercise training (AET). **(A)** Rank abundance distribution curve to visualize evenness and richness. The horizontal axis of the graph shows the OTU rank, and the vertical axis shows the relative abundance. All DZ samples represent sham group (n = 5); all MX samples indicate PD group (n = 5); all KF samples indicate AET group (n = 5). **(B–C)** Alpha diversity indices (Chao and Shannon) have no significant differences in sham, PD and AET groups. Each point indicates one sample. Pairwise comparisons of alpha and beta diversity were analyzed by the paired Wilcoxon signed rank test. **(D)** The heatmap based on binary jaccard distance analysis presented the distance of inter-group samples and that of intra-group samples to reflect the difference of microbial composition. **(E)** The PC plots revealed a separation of the samples, and no significant difference of beta diversity among three groups. Red, green, and blue indicate sham group, PD group, and AET group, respectively.

Mice were divided into three groups to subject 16S rRNA gene sequencing to investigate the changes in bacterial diversity, including sham, PD and AET mice. The raw data were uploaded to the NCBI (accession number: PRJNA797186). Results showed that a total of 3003 OTUs at 97% similarity were recognized across these 15 samples, with a maximum of 842 OTUs and a minimum of 327 OTUs. Diversity index dilution curve and Shannon curve of OTUs were plotted to evaluate the dependability of the sequencing data (Figure S1A–S1B). The results showed that dilution curve and Shannon curve for all samples attained steady values at this sequencing depth, indicating that most OUTs have been captured. Additionally, the rank abundance analysis revealed an increase of richness and a relative bacterial balance in the AET group in comparison as the PD group (Figure 3A). To estimate the differences in bacterial diversity caused by AET, sequences were aligned to assess alpha diversity and beta diversity. No significant difference in the Chao value that indicated community richness was discovered among sham, PD and AET groups (Figure 3B). Similarly, there were also no significant differences in the Shannon that represented the community diversity (Figure 3C). The heatmap based on binary jaccard distance analysis showed the distance of inter-group samples and that of intra-group samples for reflecting the difference of beta diversity (Figure 3D). Principle component analysis (PCA) showed no significant change in beta diversity in pairwise comparisons among groups (Figure 3E). Collectively, AET had no significant effect on diversity, richness, and microbial composition in feces from PD mice.

### 3.4. Alterations in the composition of fecal microflora associated with AET

**Figure 4:**
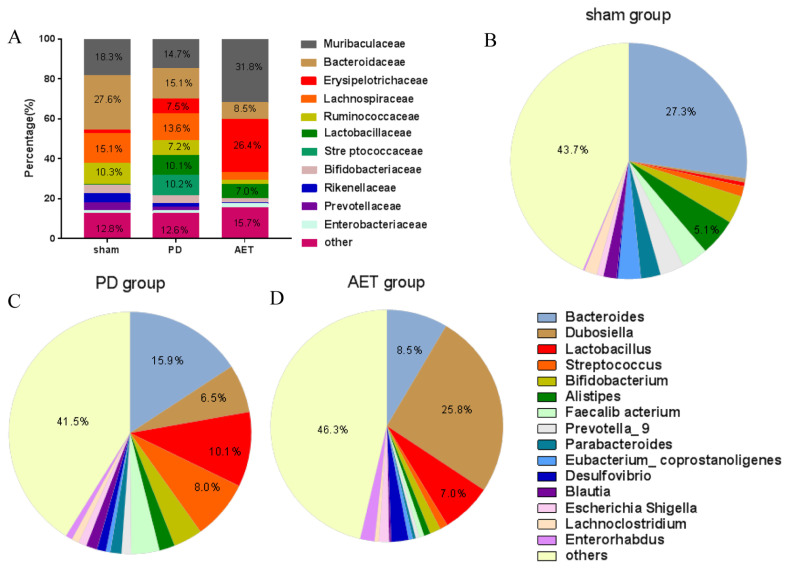
Composition proportions of fecal microbiota of the families and genus in sham group, PD group and AET group. **(A)** Component proportion of bacterial family in each group. Families with a composition greater than 0.1% are shown in the Figures **(B–D).** Component proportion of microbiota genus in sham (B), PD (C) and AET groups (D). The relative abundance of the top 15 genus is shown in the Figure. Others including all genus except the top 15.

**Figure 5:**
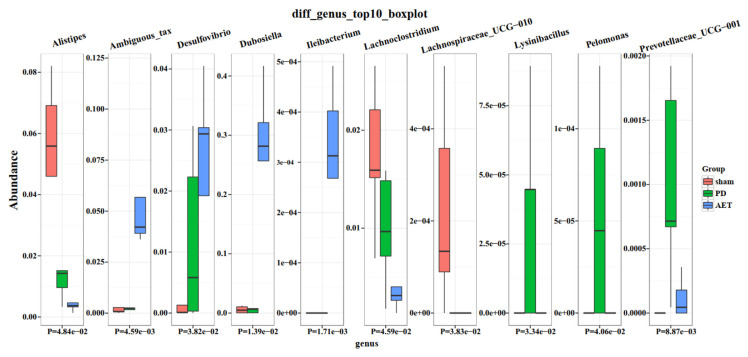
Abundance of the top 10 differentially genus in sham, PD and AET groups.

To investigate whether AET could affect gut microbiota, the proportion of microbiota at each taxon level of the phylum, family, and genus was compared among three groups. *Bacteroidetes, Firmicutes, Actinobacteria* and *Proteobacteria* were the dominant phyla in sham, PD and AET groups (Figure S2). The composition proportion of the *Bacteroidetes* was reduced, and *Firmicutes* were increased in fecal samples from PD mice compared to those of sham mice whereas AET partly rescued their relative abundance. The *Firmicutes/Bacteroidetes* ratio increased significantly in AET group. Moreover, *Muribaculaceae*, *Bacteroidaceae*, *Erysipelotrichaceae*, *Lachnospiraceae*, and *Ruminococcaceae*, *Lactobacillaceae* were predominant families. The composition proportion of *Erysipelotrichaceae* and *Enterobacteriaceae* was highest in AET group, followed by the PD group and sham group. The ratio of *Bacteroidaceae*, *Lachnospiraceae*, *Ruminococcaceae*, *Rikenellaceae*, and *Prevotellaceae* was lowest in AET group, followed by the PD group and sham group. The proportion of *Lactobacillaceae* and *Streptococcaceae* increased in PD group compared to the sham group, whereas AET partly rescued the relative abundance of the two families (Figure 4A, Table S1). Among these families, only *Rikenellaceae* in relative abundance showed statistical difference inter-group samples (data not shown). At the genus level, *Bacteroides* was the predominant genera of sham group. *Bacteroides*, *Dubosiella*, *Lactobacillus*, *Streptococcus* were the predominant genus of PD group. *Bacteroides*, *Dubosiella*, *Lactobacillus* were the predominant genus of AET group. The microbial community proportions of *Dubosiella*, Desulfovibrio, and *Escherichia-Shigella* were highest in AET group, followed by PD group and sham group. The microbial proportions of *Bacteroides*, *Alistipes*, *Prevotella_9*, *Parabacteroides*, *Eubacterium coprostanoligenes* and *Blautia* were lowest in the AET group, followed by PD group and sham group. An increase of *Lactobacillus*, *Streptococcus* and *Lactococcus* in proportion was detected in PD group compared with sham group, while AET partly abolished the alteration (Figures 4B–4D, Table S2). Additionally, Figure 5 depicted the top 10 genus with statistical difference among three groups (p < 0.05). Among these genera, the increased abundance of *Lysinibacillus*, *Pelomonas*, and *Prevotellaceae_UCG–001* in the PD group was reduced by AET. The relative abundance of *Lactobacillus_gasseri* and *uncultured_Erysipelotrichales_bacterium* significantly increased in the AET group compared with sham and PD groups (Figure S3). These results collectively illustrated that intestinal microbiome disorders caused by PD can be partially ameliorated by AET via adjusting the relative abundance and proportion of microbiota, but not diversity, richness, and composition.

### 3.5. Predicted functional composition using LEfSe analysis

**Figure 6:**
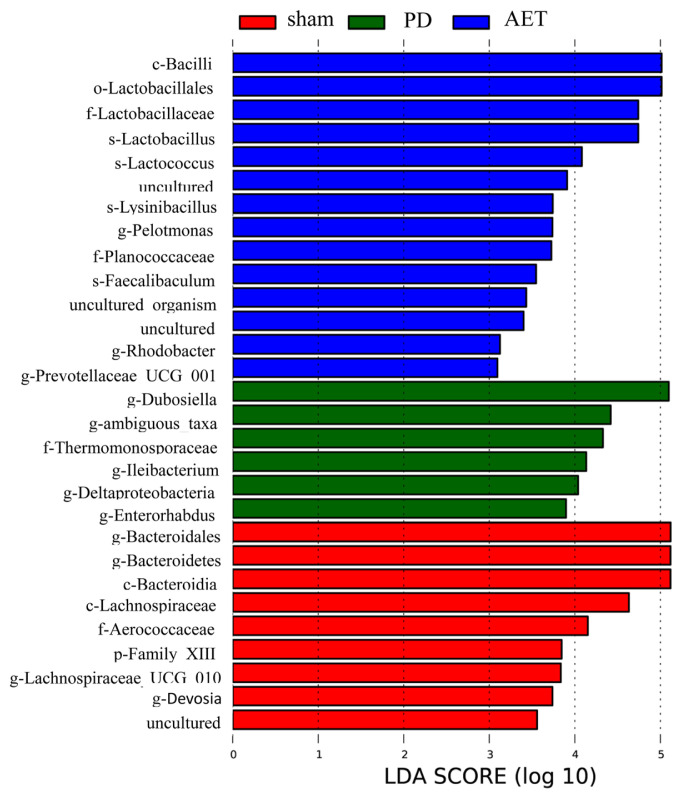
Linear discriminant analysis (LDA) integrated with effect size (LEfSe) revealed the discriminative taxon in sham, PD, and AET groups. The histogram presents the LDA score. The higher the LDA score, the greater the influence of species abundance on the difference effect. LEfSe, linear discriminant analysis effect size.

Given that this discriminant analysis did not distinguish the dominant bacteria, LEfSe was used to identify the specific taxon related with AET (Figure 6). We identified 29 discriminative bacteria. *Bacteroidales, Bacteroidetes, Lachnospiraceae_UCG_01*0 and *Devosia* were identified as specific genera in the sham group. *Dubosiella*, *ambiguous_taxa, Ileibacterium, Deltaproteob acteria*, and *Enterorhabdus* were all significantly overrepresented in the PD group, and *Bacilli*, *Lactobacillales*, *Lactobacillaceae, Lachnospiraceae, Lactobacillus*, and *Lactococcus* (LDA scores log10 > 4) were identified as specific taxon in the AET group.

## 4. Discussion

Exercise therapy is regarded a popular physical therapy, which has been developed for rehabilitation management of PD nearly 25 years (Mak et al., 2017). Many studies have documented that long-term aerobic exercise could relieve PD progression (Ahlskog, 2018; Miller Koop et al., 2019). Recently, accumulating evidence have demonstrated that exercise affects the gut microbial composition and play a positive role. People who are sedentary and people who perform physical exercise have different gut microbiota (Munukka et al., 2018). In addition, exercise can impact microbial abundance in the human gut and promotes the metabolic health (Mika and Fleshner, 2016; Welly et al., 2016). However, the beneficial effects of the aerobic exercise on fecal microbiome in PD mice or patients remain unclear.

In this study, bacterial diversity had no statistical difference in PD mice compared with sham-operated mice. Previous study showed no notable differences found in diversity of fecal bacterial communities between PD and healthy subjects (Sun et al., 2021), which were consistent with this study. Previous study showed that physical activity presents associations with α-diversity and β-diversity in gut microbiome of PD (Heinzel et al., 2020). While there is no significant change in α-diversity and β-diversity in pairwise comparisons among groups in our study. We therefore speculate that α-diversity and β-diversity may be due to the period of AET and type of exercise.

In our study, *Firmicutes/Bacteroidetes* ratio decreased significantly in AET group compared with PD group. *Firmicutes/Bacteroidetes* ratio is often interpreted as a proxy for gut health. It reported that the *Firmicutes/Bacteroidetes* ratio highly associated with inflammatory conditions, while exercise could increase *firmicutes/Bacteroidetes* ratio and reduce inflammation (C. C. Evans et al., 2014; Campbell et al., 2016). Previous studies have shown that a lower abundance of *Firmicutes* and a higher abundance of *Bacteroidetes* in Alzheimer’s Disease patients suggesting a linkage between the ratio of *Firmicutes/Bacteroidetes* and neurological function (Vogt et al., 2017). Therefore, we speculated that AET may alter PD symptoms by reducing the *Firmicutes/Bacteroidetes* ratio.

The increased proportion of *Lactobacillaceae* and *Streptococcaceae* in PD mice compared to sham mice, whereas AET partly rescued the relative abundance of these. An increase of *Lactobacillus* and *Streptococcus* in proportion was detected in PD group compared with sham group, while AET partly abolished the alteration. Increased *Lactobacillaceae* are related with a severe clinical profile, including higher frequencies of cognitive impairment and PD subtype (Barichella et al., 2019) and high levels of *Lactobacillus* is related with advanced PD (Hasegawa et al., 2015). This suggests that AET may partially improve PD symptoms by regulating the proportion of *Lactobacillaceae*. Moreover, it was reported that increased *Streptococci* and their genera are positively correlated with cadaverine levels (Shaidullov et al., 2021). Cadaverine is involved in inhibiting intestinal motility in a mouse model, increased cadshrine may also contribute to promoting proinflammatory environment and gastrointestinal motor dysfunction in PD patients (Shaidullov et al., 2021). AET reduced the proportion of *Streptococcace* that could reduce cadaverine levels, suggesting that AET relief of PD symptoms by improving inflammation and gastrointestinal motility. Additionally, a study has demonstrated that the putative cellulose degrading bacteria (*genera Blautia, Ruminococcus* and *Faecalibacterium*) significantly reduced in PD group relative to healthy subjects, and the putative pathobionts (*genera Escherichia-Shigella, Enterococcus, Proteus*) were significantly increased (Li et al., 2017). *Blautia* has lower abundances in PD fecal samples, and can produce SCFA butyrate and is associated with antiinflammatory properties (Keshavarzian et al., 2015). *Escherichia-Shigella* can result in diarrhea and generate Shiga toxin, which has a negative effect on the central nervous system of rabbits and rodents (Bridgwater et al., 1955). In our study, AET decreased the level of pathogenic *Streptococcus*, not *Escherichia-Shigella*, and had no increase of the putative cellulose degrading bacteria *Blautia* and *Faecalibacterium* and decrease of *Lactobacillus* gasseri in this study. It demonstrated that AET may improve the gut microbial environment via affecting the relative abundance of *Streptococcus*. Exercise-induced microbiota alterations may be associated with harmful protein degradation products and prolonged colon transport (Roager et al., 2016).

We found *Lysinibacillus*, *Pelomonas*, and *Prevotellaceae_UCG* got back to the normal level after AET training in our study. As a gram-positive bacterium, *Lysinibacillus* has been regarded as an environmental pollutant in earlier times (Gao et al., 2022). In the recent year, it was found that *Lysinibacillus sphaericus* impairs the chemoprophylaxis efficacy of aspirin on colorectal cancer (Zhao et al., 2020). It was reported *Pelomonas* and *Prevotella* were enriched in the endometrial cancer patients compared with the normal (Yan et al., 2021). In addition, *Prevotellaceae_UCG* promotes the production of short-chain fatty acids, improves intestinal microbial composition, regulates the immune regulatory activity related to intestinal flora, and maintains intestinal homeostasis (Xie et al., 2020). The relationship between *Lysinibacillus*, *Pelomonas*, and *Prevotellaceae_UCG* and PD occurrence and development has not been reported. According to the sequencing results, we speculated that AET might improve PD by reducing the abundance of *Lysinibacillus*, *Pelomonas*, and *Prevotellaceae_UCG*.

Analysis the specific taxon related with AET, *Bacilli, Lactobacillales, Lactobacillaceae, Lactobacillus* and *Lactococcus* were identified as specific taxon in the AET group. The above reports in combined with our study suggested that AET may improve the gut microbial environment via affecting the relative abundance of *Lactobacillus* and *Streptococcus*.

## 5. Conclusion

In summary, our study suggests that AET could change the composing proportion of gut microbiota in PD mice. AET decreased the level of potential pathogenic *Streptococcus, Lactobacillaceae*, and the ratio of *Firmicutes/Bacteroidetes* in PD mice. We elucidated discriminative bacteria of fecal microbiomes, such as *Bacilli, Lactobacillales, Lactobacillaceae, Lactobacillus*, and *Lactococcus* in the AET mice. Our study reveals that aerobic exercise may improve PD via regulating gut microbiota.

## Supplemetary Files

Figure S1 The validation of 16S rRNA sequencing data analysis. (A) Diversity index rarefaction curve assessing the number sequences. (B) Rarefaction curve for Shannon index evaluating the relative bacterial richness of the number sequences.

Figure S2 Composition proportions of fecal microbiota of the phyla in sham-operated mice, PD mice, mice receiving AET.

Figure S3 Abundance of top 10 species in sham, PD and AET groups.

**Table S1:** Component proportion of bacterial family in sham, PD and AET groups.

Gene	Sham group	PD group	AET group
other	12.80%	12.60%	15.7%
Enterobacteriaceae	1.3%	1.5%	1.8%
Prevotellaceae	4.0%	1.7%	0.2%
Rikenellaceae	4.4%	1.9%	0.4%
Bifidobacteriaceae	4.3%	3.9%	2.0%
Streptococcaceae	0.1%	10.2%	0.2%
Lactobacillaceae	0.5%	10.1%	7.0%
Ruminococcaceae	10.3%	7.2%	2.0%
Lachnospiraceae	15.1%	13.6%	4.0%
Erysipelotrichaceae	1.6%	7.5%	26.4%
Bacteroidaceae	27.3%	15.1%	8.5%
Muribaculaceae	18.3%	14.7%	31.8%

**Table S2:** Component of bacterial genus in sham, PD and AET groups. (Others represent genera that are not listed in the table).

Taxonomy	Sham group 1	Sham group 2	Sham group 3	Sham group 4	Sham group 5	PD group 1	PD group 2	PD group 3	PD group 4	PD group 5	AET group 1	AET group 2	AET group 3	AET group 4	AET group 5
Bacteroides	0,00491686	0,35316467	0,3697032	0,163507956	0,475370999	0,159038083	0,117647059	0,089308064	0,027132129	0,361523333	0,28696585	0,056275702	0,027892008	0,045592705	0,009207938
uncultured_bacterium	0,711648489	0,000804577	0,005587341	0,001028071	0,007464688	0,051716431	0,215581977	0,023779725	0,190684785	0,008269265	0,074244591	0,25563204	0,363311282	0,19578044	0,29858752
Dubosiella	4,47E-05	0,00035759	0,010593599	0,004782764	0,012560343	0,000268192	0,006481316	0,000402289	0,311371357	0,007598784	0,010951189	0,25670481	0,321383873	0,41690506	0,281557304
Other	0,16337386	0,043849455	0,048095834	0,044519936	0,060879671	0,100438048	0,145047381	0,048051135	0,037234043	0,044564634	0,07218845	0,043849455	0,037546934	0,025567674	0,032317182
Lactobacillus	0,007330592	0,000223494	0,007419989	0,002860719	0,009386733	0,057929555	0,223314858	0,023019846	0,194439478	0,005721438	0,05739317	0,027981405	0,064589666	0,127972466	0,073618809
Streptococcus	0,003441802	0,005140354	0,04331307	0,006034329	0,013230824	0,012694439	0,009476131	0,361523333	0,001609154	0,015331665	0,045503308	0,001877347	0,002503129	0,002994815	0,004335777
Bifidobacterium	0,000402289	0,129179331	0,029992848	0,016359735	0,014348293	0,027221527	0,02641695	0,016762024	0,02959056	0,093465046	0,013454318	0,008850349	0,01515287	0,02288575	0,015733953
Alistipes	0,001296263	0,069148936	0,045994994	0,082066869	0,055873413	0,009610227	0,01515287	0,014258895	0,003307706	0,064008582	0,034015734	0,004648668	0,003307706	0,003709995	0,001340962
Faecalibacterium	0,001028071	0,084793492	0,039558377	0,02641695	0,026819238	0,087475416	0,015644556	0,020337922	0,001564456	0,066869301	0,023913821	0,003575898	0,001385661	0,002011443	0,003978187
Ambiguous_taxa	0,017119614	4,47E-05	0,000670481	0,000759878	0,002771321	0,001698552	0,002369033	0,002592526	0,03803862	0,001698552	0,036116574	0,042150903	0,056767388	0,039111389	0,12113356
Prevotella_9	0,001117468	0,000223494	0,08237976	0,062175934	0,019622743	0,023556231	0,005587341	0,027668514	0,001653853	0,006570713	0,016583229	0,002056142	0,000804577	0,002145539	0,002994815
Parabacteroides	0,000134096	0,059225818	0,027087431	0,029635258	0,018281781	0,00388879	0,011308779	0,003754693	0,001162167	0,052252816	0,014124799	0,001922045	0,001028071	0,002056142	0,000223494
[Eubacterium]_coprostanoligenes_group	0,000446987	0,000759878	0,027087431	0,088190595	0,039021992	0,00670481	0,005185053	0,008716252	0,002681924	0,007196496	0,017790095	0,003441802	0,001787949	0,003933488	0,001296263
Desulfovibrio	0,007956374	0	8,94E-05	8,94E-05	0,001296263	0,005810835	0,030663329	0	0,022304667	0,000312891	0,001028071	0,019220454	0,030395137	0,029367066	0,04049705
Blautia	0,001385661	0,035356696	0,015242267	0,030797425	0,007911675	0,020203826	0,008939746	0,014303594	0,000491686	0,031378509	0,007464688	0,004425174	0,000804577	0,000581083	0,00174325
Escherichia-Shigella	0,000536385	0,002369033	0,028070803	0,002190238	0,012560343	0,015197568	0,002994815	0,015286966	0,013990703	0,006302521	0,011219381	0,054040765	0,000670481	0,001475058	0,002771321
Lachnoclostridium	0,015912748	0,01515287	0,022081173	0,006928303	0,026551046	0,009654926	0,015868049	0,001787949	0,007151797	0,014839979	0,017030216	0,004022886	0,002637225	0,003128911	0,001340962
Enterorhabdus	0,011219381	4,47E-05	0,000134096	0,000178795	0,000223494	0,005542643	0,011845164	0,001922045	0,023198641	0,000402289	0,000446987	0,010101913	0,018237082	0,032942964	0,035848382
Roseburia	0,001206866	0,000670481	0,012470946	0,037904524	0,01796889	0,033300554	0,003307706	0,01899696	0,000938673	0,004425174	0,012426247	0,001564456	0,000893975	0,001340962	0,002547828
Lachnospira	0,000670481	0,021321294	0,019935634	0,008984445	0,023153942	0,02288575	0,006436617	0,006079027	0,001340962	0,01725371	0,012292151	0,001966744	0,000893975	0,002056142	0,000804577
Lactococcus	0,002860719	0	0,00107277	0,000178795	0,00107277	0,002726623	4,47E-05	0,089352762	0,00174325	0,016940819	0	0	0	0	0,000134096
Agathobacter	0,000759878	0,00210084	0,007777579	0,009565528	0,00952083	0,023422135	0,000759878	0,013811908	0,000581083	0,004469873	0,00527445	0,00071518	0,000581083	0,000581083	0,000849276
Barnesiella	0,000134096	4,47E-05	0,00071518	0,061728947	0,002681924	0,00317361	0,000134096	0,003128911	4,47E-05	0,000178795	0,001206866	0,000134096	0,000134096	0,00035759	0,000446987
Clostridium_sensu_stricto_1	0,001340962	0,000178795	0,001564456	0,001162167	0,00174325	0,026595745	0,001028071	0,024047917	4,47E-05	0,000268192	0,001609154	0,009073842	0,000536385	8,94E-05	0,003307706
uncultured_Bacteroidales_bacterium	0,009297336	0	0	0	0	0,001251564	0,018237082	0,001028071	0,02534418	0,000312891	0,000491686	0,002681924	0,004648668	0,003263007	0,005229751
Odoribacter	0,000134096	0,025165385	0,002860719	0,012113356	0,000893975	0,00035759	0,004067584	0,001653853	0,000312891	0,021589487	0,001698552	0,00035759	4,47E-05	8,94E-05	0,000312891
Parasutterella	0,001653853	0,01935455	0,005095655	0,001966744	0,00317361	0,006794207	0,004380476	0,002994815	0,000491686	0,017432505	0,00281602	0,00035759	0,003039514	0,000536385	0,001028071
Lachnospiraceae_NK4A136_group	0,00107277	0,000536385	0,007196496	0,006391918	0,000312891	0,019488647	0,008984445	0,009476131	0,001206866	0,00035759	0,002056142	0,005676739	0,004156982	0,000312891	0,001653853
Ruminococcus_1	0,000312891	0,000134096	0,009073842	0,006257822	0,019980333	0,004559271	0,002592526	0,007554085	0,000983372	0,003620597	0,00809047	0,001832648	0,001028071	0,001340962	0,000938673
Anaerostipes	0,000178795	0,015823351	0,00563204	0,005229751	0,005900232	0,008403361	0,002950116	0,002681924	0,000223494	0,011800465	0,002905417	0,00071518	0,000223494	0,000446987	0,000804577
uncultured	0,00245843	0,00210084	0,002994815	0,000938673	0,00174325	0,008358663	0,010682997	0,007330592	0,001340962	0,003263007	0,014124799	0,000759878	0,000849276	0,000625782	0,001430359
Erysipelotrichaceae_UCG-003	0,000223494	0,000223494	0,001475058	0,034328625	0,005721438	0,006391918	0,000759878	0,005006258	0,000312891	0,00071518	0,002324334	0,000178795	0,000312891	0,000268192	0,000446987
Subdoligranulum	0,000446987	0,009476131	0,002771321	0,018237082	0,005542643	0,00245843	0,001653853	0,005006258	0,000178795	0,008492759	0,000938673	0,000312891	0,000178795	0,000268192	0,000581083
Fusicatenibacter	0,000268192	0,009565528	0,004738065	0,009699625	0,007285893	0,005006258	0,001653853	0,002011443	0,000402289	0,008671554	0,004693367	0,000446987	0,00035759	0,000223494	0,000491686
[Eubacterium]_hallii_group	8,94E-05	0,015912748	0,002011443	0,006570713	0,002145539	0,007554085	0,002056142	0,001609154	8,94E-05	0,011398176	0,001966744	0,000178795	0,000223494	0,000223494	0,000938673
Candidatus_Arthromitus	0,000938673	4,47E-05	0,011353478	0,002369033	0,005587341	0	0,000759878	0,00174325	0,00071518	0,001340962	0,022930449	0,001922045	0,001206866	0,000983372	0,000581083
[Ruminococcus]_torques_group	0,000446987	0,004872162	0,003128911	0,003397104	0,006168425	0,010504202	0,001430359	0,007777579	0,000178795	0,003978187	0,004156982	0,000536385	0,000446987	0,000670481	0,001251564
Paraprevotella	0	4,47E-05	0,00035759	0,043044878	0	0	0	0,000759878	8,94E-05	4,47E-05	0,000223494	4,47E-05	4,47E-05	0	0
Ruminococcus_2	0,000402289	0,000268192	0,001340962	0,01090649	0,002637225	0,018415877	0,000402289	0,004559271	8,94E-05	0,000312891	0,000893975	0,000178795	4,47E-05	0,000134096	0,001162167
Dorea	0,000223494	0,008001073	0,00174325	0,010325407	0,002324334	0,004022886	0,001296263	0,004156982	0,000178795	0,005855534	0,001162167	0,000223494	0,000402289	0,000670481	0,000178795
[Eubacterium]_eligens_group	0,000178795	8,94E-05	0,007554085	0,001251564	0,011532272	0,000536385	0,001385661	0,003799392	0,00071518	0,001787949	0,007285893	0,001832648	0,000536385	0,000983372	4,47E-05
Faecalibaculum	4,47E-05	0	4,47E-05	8,94E-05	4,47E-05	0	4,47E-05	0,000268192	0,02042732	0,000268192	0,00035759	0,004335777	0,004648668	0,004335777	0,004335777
Klebsiella	0,000178795	0,000223494	0,00107277	0,000849276	0,00071518	0,005810835	0,000625782	0,006436617	8,94E-05	0,000536385	0,005676739	0,009073842	0,004693367	0,000178795	0,000446987
Ruminococcaceae_UCG-002	8,94E-05	0,001206866	4,47E-05	0,02780261	0	0,004201681	0,000178795	0	4,47E-05	0,001117468	0,00035759	0,000178795	4,47E-05	0	0,000581083
Coriobacteriaceae_UCG-002	0,000446987	0	8,94E-05	0	0,000134096	0,000178795	0,001832648	0,00035759	0,00598963	8,94E-05	8,94E-05	0,001519757	0,002860719	0,01090649	0,010861792
Collinsella	8,94E-05	0,003754693	0,002369033	0,013543715	0,001117468	0,004514572	0,000581083	0,002637225	4,47E-05	0,003039514	0,000581083	4,47E-05	4,47E-05	0	0,00035759
Butyricimonas	8,94E-05	0,011889862	0,002771321	0,0035312	0,00071518	0,000759878	0,000983372	0	4,47E-05	0,010414804	0,00107277	4,47E-05	8,94E-05	8,94E-05	0,000178795
Bacillus	8,94E-05	0	0	4,47E-05	0,000312891	0,000178795	0,000223494	0,001340962	8,94E-05	0,000134096	0,000402289	0,028651886	0,000178795	0	0
Muribaculum	0,007330592	0	4,47E-05	0	8,94E-05	0,000223494	0,005095655	0,000893975	0,002592526	0,000402289	0,001385661	0,002234937	0,006034329	0,002234937	0,002637225
Clostridioides	0	0,000134096	8,94E-05	0	0	0	0	0	4,47E-05	0,000268192	0	0,02923297	4,47E-05	0	0
[Eubacterium]_ventriosum_group	4,47E-05	0,000804577	0,001028071	0,021723583	0,000312891	0,002860719	0,000178795	0,000491686	0	0,000804577	0,000312891	8,94E-05	8,94E-05	8,94E-05	0,000759878
Ruminococcaceae_UCG-014	0,001296263	0,000312891	0,000581083	0,002771321	0,00174325	0,009252637	0,000670481	0,003486501	0,004961559	0,000178795	0,002234937	0,000178795	0,000312891	0,000268192	0,000536385
Citrobacter	0,000178795	0,000134096	0,00210084	0,00035759	0,002860719	0,002994815	0,000581083	0,002190238	0,000268192	0,000804577	0,004648668	0,007196496	0,000804577	0,000268192	0,00107277
Dialister	8,94E-05	4,47E-05	0,002860719	0,010146612	0,001832648	0,006168425	0,000134096	0,002771321	8,94E-05	0,000625782	0,000804577	0,000223494	0,000134096	0,000178795	0,000178795
Helicobacter	0,00071518	4,47E-05	0,001519757	0,000670481	0,002011443	0,015421062	0,000983372	0,000804577	8,94E-05	0,000849276	0,000849276	0,000312891	0,000178795	0,00035759	0,000446987
Butyricicoccus	8,94E-05	0,006257822	0,002369033	0,001206866	0,002771321	0,000178795	0,003754693	0	0,000268192	0,005900232	0,001206866	0,000268192	4,47E-05	0,000268192	0
Alloprevotella	0,000402289	8,94E-05	0,008671554	0,000446987	0,00107277	0,004782764	0,001117468	0,001296263	0,001162167	0,000759878	0,001877347	8,94E-05	0,000491686	0,000759878	0,000491686
Pseudomonas	0,000402289	0	0,000670481	0,000268192	0,000849276	0,001564456	0	0,006526015	0,00035759	0,00107277	0,006436617	0,004559271	0,000178795	0,000134096	0
[Eubacterium]_ruminantium_group	4,47E-05	0,000625782	0,002905417	0,008358663	0,002279635	0,002011443	0,000446987	0,000759878	0,000312891	0,000759878	0,002637225	0,000223494	8,94E-05	0,000625782	0,000223494
Romboutsia	8,94E-05	0,00035759	0,005140354	0,002056142	0,001296263	0,009073842	0,000312891	0,000446987	8,94E-05	0,000670481	0,000536385	0,000268192	0,000312891	0,000402289	0,000134096
[Ruminococcus]_gnavus_group	4,47E-05	0,004201681	0,001162167	0,000849276	0,000536385	0,004335777	0,002324334	0,001162167	0	0,00245843	0,003575898	4,47E-05	4,47E-05	0,000134096	0,000268192
Pantoea	0,000178795	0	0,00107277	0,000223494	0	0,001162167	4,47E-05	0,000849276	4,47E-05	4,47E-05	0,000446987	0,016002146	0	0	4,47E-05
Rodentibacter	0	0	0,000491686	0,000223494	0,001564456	0	4,47E-05	0,002860719	0	0,000134096	0,014750581	0	0	0	0
uncultured_organism	0,000134096	0	8,94E-05	0	4,47E-05	0,001698552	0,01090649	0,001698552	0,002279635	4,47E-05	0	0	0	0	0,000134096
Lachnospiraceae_ND3007_group	0	0,001832648	0,001251564	0,001385661	0,00527445	0,001162167	0,000625782	0,000446987	0,000312891	0,002279635	0,001117468	0,000402289	0,000402289	0,000402289	8,94E-05
Carnobacterium	0	0	0	4,47E-05	8,94E-05	0	4,47E-05	0	0	0	0	0,016762024	0	0	0
Sutterella	0,000312891	0	0,002994815	0,000268192	0,002637225	0,004514572	0,00107277	0,000938673	0,000268192	0,000938673	0,001340962	0,000446987	4,47E-05	8,94E-05	0,000446987
Weissella	4,47E-05	0,00035759	0,000581083	8,94E-05	0,000312891	0,001117468	0	0,000491686	0	0,000312891	0	0,012649741	0,000134096	4,47E-05	0
Enterococcus	0	0	0,000134096	0,000134096	0,000849276	0,006213124	0,000268192	0,000446987	4,47E-05	0,000268192	0,003620597	0,002190238	0	0	0
Staphylococcus	4,47E-05	0	0,000938673	0,000134096	0,000312891	4,47E-05	0	8,94E-05	0,000223494	0	4,47E-05	0,011085285	4,47E-05	0	0
Fusobacterium	0	0,000581083	0,000491686	0,000849276	0,000178795	0,000536385	0,003441802	0,001028071	0	0,002145539	0,00174325	0,001162167	0,000268192	4,47E-05	0
Bilophila	0,000134096	0,004335777	0,001296263	0,000491686	0	0,000223494	0,000983372	0	0,000312891	0,003352405	0,000491686	4,47E-05	8,94E-05	8,94E-05	0,000223494
Rikenellaceae_RC9_gut_group	0,000268192	0	0	0	0,000983372	0,006928303	0,000268192	0,001430359	0,000134096	0,000268192	0,000893975	0,000178795	0	4,47E-05	0,000625782
Turicibacter	0,000223494	8,94E-05	0,002011443	0,001162167	0,001698552	0,002503129	0,000312891	0,001653853	8,94E-05	0,000625782	0,00107277	8,94E-05	0,000178795	0	8,94E-05
Ruminiclostridium_5	0,000134096	0,004380476	0,001564456	0,000804577	0	4,47E-05	0,001162167	0	4,47E-05	0,002681924	0	8,94E-05	4,47E-05	0	0,000178795
Prevotellaceae_NK3B31_group	0	0	0,00035759	8,94E-05	0	0,001206866	0	0	0,008984445	0	4,47E-05	0	0	0	0,000134096
Eggerthella	0	0,00491686	0,001385661	0,000312891	0	0	0,000670481	0	0	0,002994815	0	0	0	0	0
Ruminococcaceae_UCG-005	8,94E-05	0,000178795	0,000804577	0,002413731	0,000134096	0,001475058	8,94E-05	0,003754693	4,47E-05	0,000223494	0,000223494	0	0,000134096	0	0,000581083
Christensenellaceae_R-7_group	0,000178795	0	0,000402289	0,006302521	0,000938673	4,47E-05	8,94E-05	0,000938673	0	0,000178795	0,000312891	0	8,94E-05	4,47E-05	0,000446987
Sphingomonas	4,47E-05	8,94E-05	0	0	4,47E-05	0,001698552	0	0,002681924	0,000134096	0,000446987	0,003978187	0,000134096	4,47E-05	4,47E-05	0
Brevundimonas	0	0	8,94E-05	0	4,47E-05	8,94E-05	0	0,007822278	0	0,000804577	0,00035759	4,47E-05	0	0	0
Lachnospiraceae_UCG-001	0,000178795	4,47E-05	0,001296263	4,47E-05	0,000804577	0,002011443	0,002145539	0,000804577	4,47E-05	0,000223494	0,000759878	8,94E-05	4,47E-05	8,94E-05	0,000223494
Prevotella_2	8,94E-05	0	0,003844091	0,000178795	0,001922045	0	0,000849276	0	4,47E-05	0,000625782	0,000670481	0,000178795	4,47E-05	0,000178795	0
Oscillibacter	4,47E-05	0,002234937	0,000849276	0,000849276	0,000223494	0,000893975	0,000938673	0,000268192	0	0,001787949	8,94E-05	0,000134096	0	0,000134096	8,94E-05
Ruminiclostridium_9	8,94E-05	0,00174325	0,000983372	0,001475058	0,000223494	0	0,000312891	0,000491686	0	0,001385661	0,001296263	8,94E-05	4,47E-05	4,47E-05	0,000134096
Lachnospiraceae_UCG-006	0,001028071	0	0,000134096	0	0	0,006302521	0,000178795	0	0,000134096	4,47E-05	4,47E-05	4,47E-05	0,000223494	8,94E-05	4,47E-05
Erysipelatoclostridium	0,000223494	0,000670481	0,000625782	0,000178795	0,000223494	0	0,000312891	0	0	0,000491686	0	0,005229751	4,47E-05	0	0
Haemophilus	0	0,00071518	0,000849276	0,000983372	0,000581083	0,001475058	0,000491686	0,000759878	4,47E-05	0,000178795	0,001430359	0,000223494	8,94E-05	8,94E-05	0
Ruminococcaceae_UCG-013	8,94E-05	0,002413731	0,000625782	0,000134096	0	0	0,000625782	0,00107277	0,000268192	0,001519757	0,000804577	0	0,000134096	0	0
Hypnocyclicus	0	0	0	0	0	0	0	0	0	0	0,000312891	0	0	0	0,007241194
Coprococcus_3	0	0,001117468	0,00071518	0,002547828	0	0	0,000178795	0,001877347	0	0,000893975	0	0	8,94E-05	0	4,47E-05
Coprobacter	4,47E-05	4,47E-05	0,000223494	0,001340962	0,000938673	0,000804577	8,94E-05	0,002771321	0	0,000402289	0	4,47E-05	4,47E-05	0,000134096	4,47E-05
Gemmatimonas	0	0	0,00035759	0	0,000134096	0,000536385	0	0	0	0	0,005721438	0	0	0	0
Holdemanella	0	0	0,000178795	4,47E-05	0,000670481	0,004156982	0,000134096	0	4,47E-05	0,00035759	0,000849276	0	0	0	0,000223494
Acetobacter	0,000223494	4,47E-05	0	0	0	0,001877347	0	0,003352405	0	4,47E-05	0	0,000223494	0	0	0,000402289
Nocardioides	0	0	0	8,94E-05	0	0	0	0	0	0	0,005676739	0	0	0	0,000312891
Stenotrophomonas	0	0	0,000268192	0	0	0,002905417	0	0,000759878	0	0,000312891	8,94E-05	0,001430359	4,47E-05	0	0
Prevotellaceae_UCG-001	4,47E-05	0	0	0	0	0,001653853	0,000670481	0,001922045	0,00071518	4,47E-05	0,00035759	0	0,000178795	0	4,47E-05
[Eubacterium]_xylanophilum_group	0,000268192	4,47E-05	0,000312891	4,47E-05	0,000402289	0,002905417	0,000268192	0	0	0,000178795	0,000134096	0,000178795	0,000134096	8,94E-05	0,000178795
Arcobacter	0	0	0	0	0	0	0	0	0	0	0	0	0	0	0,005006258
Mycoplasma	0,001787949	0	0	0,000134096	0,000581083	4,47E-05	0	0,000938673	0,000223494	0,000402289	0,000536385	4,47E-05	8,94E-05	0	4,47E-05
Coprococcus_2	8,94E-05	0	0,000536385	0,001922045	0,000134096	0,001922045	0	0	0	0	0	4,47E-05	4,47E-05	0	8,94E-05
Ruminococcaceae_NK4A214_group	4,47E-05	0	0	0,001340962	0	0,001877347	0	0,000625782	0	0	0,000491686	4,47E-05	4,47E-05	0	0,000134096
Gordonibacter	4,47E-05	0,000893975	0	0	0,00035759	8,94E-05	4,47E-05	0	0	0,000670481	4,47E-05	0,000312891	0,000402289	0,000893975	0,000670481
Mitsuokella	0	0	0,000402289	0	0	0,003754693	0	0	0	0	0,000134096	0	4,47E-05	0	0
Prevotella_1	0,000134096	0	0,000402289	0	0	0,001832648	4,47E-05	0	0	0,000134096	0,001028071	0,000134096	0	0	0,000178795
Delftia	0	0	0	4,47E-05	0	0,000536385	0	0,002860719	0	0,000312891	0	0	0	0	0
Tyzzerella_3	0	0,000625782	0,000581083	0,000178795	0,000983372	4,47E-05	4,47E-05	0	0	0,000625782	0,00035759	0,000134096	0	4,47E-05	0
Flavonifractor	8,94E-05	0,000759878	0,000536385	0,000491686	0	0,000893975	0,000178795	0	4,47E-05	0,000134096	0,000268192	0,000223494	0	0	0
Marvinbryantia	0,000759878	0	8,94E-05	0,000446987	4,47E-05	0,001296263	0,000402289	0	8,94E-05	0,000134096	8,94E-05	4,47E-05	4,47E-05	4,47E-05	0
Prevotellaceae_UCG-003	0,000134096	0,000134096	0,000670481	0	0	0,002324334	0	0	0	0	8,94E-05	0	8,94E-05	0	0
Bosea	0	0	0,000625782	8,94E-05	0,000178795	0,000268192	8,94E-05	0,001340962	0	0,000134096	0,000491686	0	0	4,47E-05	8,94E-05
Parvibacter	0,000223494	0	0	0	0	4,47E-05	0,000178795	0	0,000491686	0	0	0,000134096	0,000178795	0,000893975	0,001162167
Succinivibrio	0	4,47E-05	0,001475058	0,000134096	0	0	0,00035759	0,00107277	0	4,47E-05	0	8,94E-05	0	0	0
Bryobacter	0	0	0	0	0	0,002056142	0	0	4,47E-05	0	0,001117468	0	0	0	0
Flavobacterium	4,47E-05	0	0	0	0,000178795	0	0	0,001206866	0	0	0,001162167	0,000402289	0	0	0,000223494
Catenibacterium	0,000134096	0	4,47E-05	4,47E-05	0	0,002905417	0	0	0	4,47E-05	0	0	0	0	4,47E-05
Haliangium	4,47E-05	0	0	0	0,000581083	0	8,94E-05	0	0	0	0,002413731	0	4,47E-05	0	0
Acidothermus	0	4,47E-05	0	0	0,000938673	0,001787949	8,94E-05	0	0	0	0,000268192	0	0	0	0
Ruminococcaceae_UCG-010	4,47E-05	0,000134096	0	0,000134096	0	0	0	0,002324334	0	8,94E-05	0,000134096	0	8,94E-05	0	0,000134096
Shewanella	0	0	0,000491686	0	0	0,001251564	0,000178795	0	8,94E-05	8,94E-05	0,000402289	8,94E-05	0,000134096	0,000312891	0
[Clostridium]_innocuum_group	0	0,000134096	0	8,94E-05	0	0	0,00071518	0	0	0,000223494	0,000223494	0,001296263	0	0	0
Ellin6067	0	0	0	0	0	0	4,47E-05	0,000178795	4,47E-05	0	0,002413731	0	0	0	0
Bradyrhizobium	4,47E-05	0	0,000312891	8,94E-05	4,47E-05	0,000983372	4,47E-05	8,94E-05	4,47E-05	0,000134096	0,00071518	8,94E-05	0	4,47E-05	4,47E-05
Lachnospiraceae_FCS020_group	4,47E-05	0	4,47E-05	0,000938673	0,000178795	8,94E-05	0,000134096	0,000581083	4,47E-05	0,000134096	0,000312891	0	4,47E-05	4,47E-05	0
Ruminiclostridium	8,94E-05	4,47E-05	0,000759878	0,000134096	0,000312891	8,94E-05	0,000581083	0	0,000134096	0	0,000223494	0	0,000134096	4,47E-05	0
Altererythrobacter	0	0	0	0	0	0	0	0,000178795	0	4,47E-05	0,002234937	8,94E-05	0	0	0
Ruminococcaceae_UCG-003	0	0,000536385	0,001162167	0,000312891	0	0	0,000134096	0	0	0,000312891	0	4,47E-05	0	0	0
Ramlibacter	4,47E-05	0	0,000223494	0	0	0	0	0	0	0	0,002190238	0	0	0	0
Rhodoplanes	0	0	0	0	0	0,001609154	0	0	0	0	0,000804577	0	0	0	0
Ruminococcaceae_UCG-011	0	0	0,000536385	0	0	0	0	0,001564456	0	0	0,000134096	0	0,000178795	0	0
Acidibacter	0	0	0	0	0	0,000983372	0	0	0	0	0,001251564	0	4,47E-05	0	0
Streptomyces	0	0	0	0	0	0	4,47E-05	0,001206866	0	0	0,001028071	0	0	0	0
uncultured_Hyphomicrobiaceae_bacterium	0	0	0	0	0	0,002190238	0	0	0	0	0	4,47E-05	0	0	0
Pseudarthrobacter	0	0	0	8,94E-05	0	0,000938673	4,47E-05	0	0	0	0,000625782	0,000491686	4,47E-05	0	0
Senegalimassilia	0	0	0,00035759	0	0	0,001698552	0	0	0	0	0,000178795	0	0	0	0
Anaeroplasma	8,94E-05	0	0	0	0	0,00174325	0	0	0	0	0	0	8,94E-05	0	0,000223494
Chryseobacterium	0	0	0	4,47E-05	0	0,000446987	0	0,001385661	0	0,000223494	4,47E-05	0	0	0	0
Flavisolibacter	0	0	0	0	0	0	0	0	0	0	0,002145539	0	0	0	0
Dongia	0,000178795	0	0	0	0,00071518	0	0	0	0	0	0,00107277	0	0	0	8,94E-05
Mycobacterium	0	0	0,00035759	4,47E-05	0	0,001162167	0	0	0	0	0,000268192	8,94E-05	0	0	0,000134096
Megasphaera	0	0	0,000893975	0,00107277	0	0	4,47E-05	0	0	0	0	0	0	0	0
Massilia	0	0	0	0	0	0,000223494	0	0	0	0	0,001698552	8,94E-05	0	0	0
Adlercreutzia	0	0	0	0,00071518	0,000268192	8,94E-05	0	0	0	0	0,000759878	0	0	0,000134096	0
Rhizobacter	0	0	0	0	0,000223494	4,47E-05	0	0	0	0	0,001653853	0	0	0	0
Coprococcus_1	4,47E-05	0	0,000268192	0,001430359	0	0	0	0	0	0	0	0	0	0	0,000134096
uncultured_Acidimicrobidae_bacterium	8,94E-05	0	0	0	0	0,00174325	0	0	0	0	0	0	0	0	0
Pseudonocardia	0	0	0	0	0	0,001475058	8,94E-05	0	0	0	0,000223494	0	0	0	0
uncultured_rumen_bacterium	8,94E-05	0	0,000178795	0	0,000268192	0,000849276	0	0	0	0,000178795	0,000178795	0	0	0	4,47E-05
Olsenella	0	0	0	0,00174325	0	0	0	0	0	0	0	0	0	0	0
A2	0,000178795	0	0,000268192	0	0	0	0,000670481	0,000491686	0	4,47E-05	4,47E-05	4,47E-05	0	0	0
CAG-56	4,47E-05	0	0,000223494	0,000983372	0,000268192	0	4,47E-05	0	8,94E-05	0	8,94E-05	0	0	0	0
Devosia	0	0	0,000134096	0,00035759	0,000446987	0,000223494	0,000134096	0,000312891	4,47E-05	4,47E-05	0	0	0	4,47E-05	0
Gaiella	8,94E-05	0	0	4,47E-05	0	0	0	0	0	0	0,001564456	0	4,47E-05	0	0
Solirubrobacter	0	0	0	8,94E-05	0,000312891	0	0	0	0	0	0,001340962	0	0	0	0
Fibrobacter	0	0	0	8,94E-05	0,000312891	0,00107277	0	0	0	0	0,000178795	0	0	0	0
GCA-900066575	0,000849276	4,47E-05	4,47E-05	0,000178795	0	4,47E-05	0,000223494	0	0,000134096	8,94E-05	0	0	4,47E-05	0	0
Actinomyces	0	0,000849276	0,000268192	4,47E-05	0	0	0,000178795	0	0	0,000268192	0	4,47E-05	0	0	0
Rhodobacter	0	0	0	4,47E-05	0	0	0,000178795	0,001162167	4,47E-05	0,000178795	0	0	0	0	0
Neisseria	0	0	0	0	0	0	0	0,001609154	0	0	0	0	0	0	0
Megamonas	0	0	0	0,001340962	0,000268192	0	0	0	0	0	0	0	0	0	0
metagenome	8,94E-05	0	0	0	0	0	4,47E-05	0	0	0	0,001430359	0	0	0	0
Anaerotruncus	0,000178795	0	0,000134096	0,000402289	0	0	4,47E-05	0,000625782	4,47E-05	0	0	0	0,000134096	0	0
Ileibacterium	0	0	0	0	8,94E-05	0	0	0	0	0	0	0,000268192	0,000491686	0,000312891	0,000402289
Intestinimonas	0,000178795	0,000312891	0	0,000178795	0	4,47E-05	0,000178795	8,94E-05	4,47E-05	0,00035759	0,000178795	0	0	0	0
Occallatibacter	0	0	0,000312891	0	0	0,001117468	0	0	0	4,47E-05	0	8,94E-05	0	0	0
Candidatus_Saccharimonas	0,000536385	0	0	0	0	0	4,47E-05	0	0,000536385	0	0	0	8,94E-05	4,47E-05	0,000268192
Actinoplanes	0	0	0,000178795	0	0	0	0	0,000581083	0	0	0,000759878	0	0	0	0
Aeromonas	0,000178795	0	4,47E-05	0	0	0,00035759	0	0,000446987	0	0	0,000223494	0,000268192	0	0	0
Phascolarctobacterium	0	0,00035759	0,000491686	0	0	0	4,47E-05	0	0	0,000536385	0	0	4,47E-05	0	0
Sphingobacterium	4,47E-05	0	0	0	0,000581083	0	0	0,000178795	0	4,47E-05	0,000223494	0,000402289	0	0	0
Cellulosimicrobium	0	0	0	0	0	0,000849276	0	0,000491686	0	0	0	4,47E-05	0	0	0
Phenylobacterium	0	0	0	0	0	0	0	0,000134096	0	0	0,001251564	0	0	0	0
Serratia	0	0	0	0	0,000893975	0	8,94E-05	0	0	0	0	0,000402289	0	0	0
Acinetobacter	4,47E-05	0,000134096	8,94E-05	0,000134096	8,94E-05	4,47E-05	8,94E-05	0	0	0,00035759	4,47E-05	0,000134096	4,47E-05	8,94E-05	8,94E-05
uncultured_Thermoanaerobacterales_bacterium	0	0	0	0	0	0,001340962	0	0	0	0	0	0	0	0	0
Ruminococcaceae_UCG-004	0	0,000536385	8,94E-05	0,000134096	0	0	4,47E-05	0	0	0,000446987	4,47E-05	0	0	0	0
Tyzzerella	0	0,000446987	0,000223494	0	0,000134096	0	8,94E-05	0	0	0,000312891	0	4,47E-05	4,47E-05	0	0
Rikenella	0,000625782	0	0	4,47E-05	0	0	0	0	0,000536385	0	0	0	0	4,47E-05	0
Rhizocola	0	0	0	0	0,000223494	0,001028071	0	0	0	0	0	0	0	0	0
Lachnospiraceae_UCG-010	8,94E-05	0,00035759	0,000134096	0,000536385	0	0	0	0	0	0,000134096	0	0	0	0	0
Georgenia	0	0	0	0	8,94E-05	0	0	0,000893975	0	0	0,000268192	0	0	0	0
Globicatella	0	0	0,000268192	0,000178795	0,000759878	4,47E-05	0	0	0	0	0	0	0	0	0
Faecalitalea	0	0,000134096	0,00035759	0,00035759	0	0	0	0	4,47E-05	0,000134096	0,000178795	0	0	4,47E-05	0
Allorhizobium-Neorhizobium-Pararhizobium-Rhizobium	0	0	0,000178795	8,94E-05	0,000223494	0	0,000134096	4,47E-05	0	4,47E-05	0,000178795	0,000223494	0	8,94E-05	0
Acetitomaculum	0	0	0	0	0	0,001206866	0	0	0	0	0	0	0	0	0
Veillonella	0	0,000134096	0	0	0	0	0	0	0	0	0	0,001028071	0	0	0
Proteus	0	0	4,47E-05	0	0	0,000983372	0,000134096	0	0	0	0	0	0	0	0
Hungatella	0	0,000178795	0	0,000178795	0	0	0,000268192	0	0	8,94E-05	0,000446987	0	0	0	0
Anaeromyxobacter	0	0	0	0	0	0	0	0,000536385	0	0	0,000536385	0	0	0	4,47E-05
Lysobacter	8,94E-05	0	0	0	0	0	0	0	0	4,47E-05	0,000983372	0	0	0	0
Phaselicystis	0	0	0	0	0	0	0	0	0	0	0,00107277	0	0	0	0
Ochrobactrum	0	0	8,94E-05	4,47E-05	4,47E-05	0,000581083	4,47E-05	0,000134096	0	0	0,000134096	0	0	0	0
Family_XIII_AD3011_group	0	0,000178795	0	0,000670481	0	0	4,47E-05	0	0	0,000178795	0	0	0	0	0
Allisonella	0	0,000134096	0,00035759	0	0	0	0,000134096	0	8,94E-05	0,000134096	0	8,94E-05	8,94E-05	4,47E-05	0
Defluviicoccus	0	0	0	0	0	0,001028071	0	0	0	0	0	0	0	0	0
uncultured_Porphyromonadaceae_bacterium	0	0	0,001028071	0	0	0	0	0	0	0	0	0	0	0	0
Slackia	0	0	0	0	0,000893975	0	8,94E-05	0	0	0	0	0	0	0	0
Brumimicrobium	0	0	0	0	0	0,000983372	0	0	0	0	0	0	0	0	0
Hamadaea	0	0	0	0	0	0	0	0	0	0	0,000983372	0	0	0	0
Cetobacterium	0	0	0	0	0	0	0	0,000446987	0	0	0,000491686	0	0	0	0
Blastococcus	0,000134096	0	0,000134096	0	0	0	0	0	4,47E-05	0	0,000625782	0	0	0	0
GCA-900066225	0	0	0	0,000446987	0,000446987	0	0	0	0	0	0	0	0	0	0
Novosphingobium	4,47E-05	0	0,000134096	0	8,94E-05	0	0	0	0	0	0,000178795	0,000446987	0	0	0
Variovorax	0	0	0	0	0	0	0	0	0	0	0,000893975	0	0	0	0
Sphingobium	0	0,000134096	0	0	0	0	0	0	0	0,00035759	0	0,000402289	0	0	0
Papillibacter	0	0	0	0	0,000849276	0	0	0	0	0	0	0	0	0	0
Rhodococcus	0	4,47E-05	0	0	0	0	0	0,000670481	0	8,94E-05	0	0	0	0	0
UBA1819	0	0	0	0,000625782	0	0	0,000178795	0	0	0	0	0	0	0	0
uncultured_gamma_proteobacterium	0	0	0	0	0	0,000804577	0	0	0	0	0	0	0	0	0
Amycolatopsis	0	0	0	0	0	0	0	0,000804577	0	0	0	0	0	0	0
Candidatus_Soleaferrea	0	0	0	4,47E-05	0,000625782	0	0	0	0	8,94E-05	0	0	0	0	0
Acetatifactor	4,47E-05	4,47E-05	0	0	0	0,000670481	0	0	0	0	0	0	0	0	0
Chryseolinea	8,94E-05	0	0	0	0	0	0	0	0	0	0,000670481	0	0	0	0
Desulfobacca	0	0	0	0	0	0	0	0,000759878	0	0	0	0	0	0	0
Tyzzerella_4	0	8,94E-05	0	0	0	4,47E-05	0,000268192	0	0	8,94E-05	0,000268192	0	0	0	0
Arenimonas	0	0	0	0	0	0	0	0	0	0	0,00071518	0	0	0	0
[Eubacterium]_brachy_group	4,47E-05	0	0	0,000268192	0	4,47E-05	8,94E-05	0	0,000134096	8,94E-05	0	0	4,47E-05	0	0
Nonomuraea	4,47E-05	0	0	0	0	0	0	0	0	0	0,000625782	0	0	0	0
Rubrobacter	0	0	0	0	0	0	0,000134096	0	0	0	0,000536385	0	0	0	0
Granulicella	0	0	0	0	0	0,000670481	0	0	0	0	0	0	0	0	0
Adhaeribacter	0	0	0	0	0	0	0	0	0	0	0,000670481	0	0	0	0
Nannocystis	0	0	0	0	0	0	0	0	0	0	0,000670481	0	0	0	0
Corynebacterium_1	0	0	0,000134096	4,47E-05	0,000402289	0	0	0	4,47E-05	0	0	4,47E-05	0	0	0
Sediminibacterium	0	0	8,94E-05	0	0,000268192	0,000223494	0	0	0	4,47E-05	4,47E-05	0	0	0	0
MND1	0,000134096	0	0,000178795	0	0	0	0	0	0	0	0,000312891	0	0	4,47E-05	0
uncultured_prokaryote	4,47E-05	0	0	0	0	0	4,47E-05	0,000536385	0	0	0	0	0	0	0
Rubellimicrobium	0	0	0	0	0	0	0	0	0	0	0,000625782	0	0	0	0
Actinophytocola	0	0	0	0	0	0	0	0	0	0	0,000625782	0	0	0	0
Iamia	0	0	0	0	0	0	0	0	0	0	0,000625782	0	0	0	0
Ohtaekwangia	0	0	0	0	0	0	0	0	0	0	0,000625782	0	0	0	0
Agromyces	0	0	0	0	0	0	0	0	0	0	0,000625782	0	0	0	0
Prevotella_7	0	4,47E-05	0	0	0,000491686	0	0	0	0	0	0	4,47E-05	0	0	0
Conexibacter	0	0	0	0	0	0,000581083	0	0	0	0	0	0	0	0	0
Chujaibacter	0	0	0	0	0	0	0	0	0	0	0,000581083	0	0	0	0
Thermopolyspora	4,47E-05	0	0	0	0	0	0	0	0	0	0	0	0,000491686	0	0
Azospirillum_sp._47_25	0	0	0	0	0	0	0	0,000446987	0	0	8,94E-05	0	0	0	0
Steroidobacter	0	0	0	0	0	0	0	0	0	0	0,000446987	0	0	8,94E-05	0
Ruminiclostridium_6	4,47E-05	0	0,000134096	0	0,000223494	0	4,47E-05	0	4,47E-05	4,47E-05	0	0	0	0	0
Mucispirillum	0	0	0	4,47E-05	0	0,000268192	4,47E-05	0	0	0	0	0	4,47E-05	0	0,000134096
Actinocorallia	0	0	0	0	0	0	0	0	0	0	0,00035759	0	0	0	0,000178795
Pajaroellobacter	0	0	0	0	0	0	0	0	0	0	0,000491686	0	0	0	0
freshwater_sediment_metagenome	0	0	0,000491686	0	0	0	0	0	0	0	0	0	0	0	0
mouse_gut_metagenome	0,000268192	0	0	0	0	0	0	0	8,94E-05	0	0	0	4,47E-05	0	8,94E-05
Exiguobacterium	0	0	0,000402289	0	0	4,47E-05	0	0	0	0	0	4,47E-05	0	0	0
Gemmatirosa	0	0	0	0	0	0	0	0,000178795	0	0	0,000312891	0	0	0	0
Ureaplasma	0,000268192	0	0	0	0	0	0	0	0	0	0	0	0	0	0,000223494
Candidatus_Hepatoplasma	0	0	0,000446987	0	0	0	0	0	0	0	0	0	0	0	0
Ruminococcaceae_UCG-009	4,47E-05	0	0	4,47E-05	0	0	0	0	0	0	0,000178795	0	0	0	0,000178795
Negativibacillus	0	0	0	0,000223494	0	0	0	0	0	4,47E-05	0,000134096	0	4,47E-05	0	0
Mesorhizobium	0	0	0	0	8,94E-05	0	0	0	0	0	0,00035759	0	0	0	0
Sellimonas	0	0	0	0	8,94E-05	0	0	0	0	0	0,000223494	0	0	0	0,000134096
Rhodomicrobium	0	0	0,000134096	0	0	0	0	0	0	0	0,000312891	0	0	0	0
Nitrosospira	0,000268192	0	0	0	0	0	0	0	0	0	0,000178795	0	0	0	0
Eubacterium	0	0,000178795	0	0	0	0	0	0	0	0,000134096	0	8,94E-05	0	0	0
Burkholderia-Caballeronia-Paraburkholderia	0	0	0	0	0,000402289	0	0	0	0	0	0	0	0	0	0
Sandaracinus	0	0	0	0	0	0	0	0	0	0	0,000402289	0	0	0	0
Parafilimonas	0	0	0	0	0	0	0	0	0	0	0,000402289	0	0	0	0
CL500-29_marine_group	0	0	0	0	0	0	0	0	0	0	0,000402289	0	0	0	0
Flavitalea	0	0	0	0	0	0	0	0	0	0	0,000402289	0	0	0	0
Dielma	0	0	0,000223494	0	0	0	0	0	0	0,000178795	0	0	0	0	0
uncultured_Gemmatimonadetes_bacterium	0	0	0	0	0,000402289	0	0	0	0	0	0	0	0	0	0
Caenimonas	0	0	0	0,000134096	0	0	0	0	0	0	0,000268192	0	0	0	0
Phreatobacter	0	0	8,94E-05	0	0	0	0	0	8,94E-05	8,94E-05	8,94E-05	0	0	0	0
Thermoactinomyces	0	0	8,94E-05	0	0	0	4,47E-05	0,000134096	0	8,94E-05	0	0	0	0	0
Candidatus_Solibacter	0	0	0	8,94E-05	0	0	0	0	0	0	0,000223494	0	4,47E-05	0	0
Cellulomonas	0	0	0	0	0	0	4,47E-05	0	0	0	0,000312891	0	0	0	0
unidentified	8,94E-05	0	0	0	0	0,000268192	0	0	0	0	0	0	0	0	0
Methylobacterium	0	0	0	0	0	0,000178795	0	0,000134096	0	0	4,47E-05	0	0	0	0
Dyella	0	0	0	0	0	0	0	0,00035759	0	0	0	0	0	0	0
Vulgatibacter	0	0	0	0	0	0	0	0	0	0	0	0	0	0	0,00035759
alphaI_cluster	0	0	0	0	0	0	0	0	0	0	0,00035759	0	0	0	0
Epulopiscium	0	0	0	0	0	0	0	0	0	0	0	0,00035759	0	0	0
Anoxybacillus	0	0	8,94E-05	0	4,47E-05	8,94E-05	0	0	0	0	8,94E-05	0	0	0	0
DNF00809	0	0	0	0	0	0	0	0	8,94E-05	0	0	4,47E-05	4,47E-05	0	0,000134096
Phocea	0	8,94E-05	0	8,94E-05	0	0	0	0	0	0,000134096	0	0	0	0	0
Anaerovorax	0	0	0	0	4,47E-05	0	0	0,000268192	0	0	0	0	0	0	0
Comamonas	0	0	0	0,000134096	0	0	0	0	0	4,47E-05	0,000134096	0	0	0	0
Reyranella	0	0	0	0	0	0	4,47E-05	0	0	0	0,000268192	0	0	0	0
Terrimonas	0	0	0	0	0	0	0	0	0	0	0,000312891	0	0	0	0
Subgroup_10	0	0	0	0	0	0	0	0	0	0	0,000312891	0	0	0	0
Hyphomicrobium	0	0	0	0	8,94E-05	0	0	0	0	4,47E-05	0,000134096	0	0	0	0
Holdemania	0	0,000134096	0	8,94E-05	0	0	0	0	0	4,47E-05	0	0	0	0	0
Pelomonas	0	0	0	0	0	0,000134096	4,47E-05	8,94E-05	0	0	0	0	0	0	0
Arthrobacter	0,000134096	0	0	0	0	0	4,47E-05	0	0	0	8,94E-05	0	0	0	0
Enhydrobacter	0	0	4,47E-05	0	0	0	0	0	0	0	0,000223494	0	0	0	0
Pseudolabrys	0	0	0	0	0	0	0	0	0	0	0,000223494	0	4,47E-05	0	0
Acidovorax	0	0	0,000178795	0	0	0	0	0	0	0	8,94E-05	0	0	0	0
Tahibacter	0	0	0	0	0	0	0	0	0	0	0,000268192	0	0	0	0
Lachnospiraceae_AC2044_group	0	0	0	0	0	0	0	0	0	0	0,000134096	0	0,000134096	0	0
Pediococcus	0	0	0	0	0	0	0	0,000268192	0	0	0	0	0	0	0
BIyi10	0	0	0	0	0	0	0	0	0	0	0,000268192	0	0	0	0
Curtobacterium	4,47E-05	0	0	4,47E-05	0	0	0	0	0	0	8,94E-05	4,47E-05	0	0	0
Catabacter	0	4,47E-05	0	4,47E-05	0	0	0	0	0	8,94E-05	0	0	4,47E-05	0	0
Coprobacillus	0	0	0	4,47E-05	0	0	0	0	4,47E-05	0	0,000134096	0	0	0	0
Morganella	0	0	0	4,47E-05	0	0	0	0	0	0	0	0	0,000178795	0	0
DTU089	0	8,94E-05	0	0	0	0	0	0	0	0,000134096	0	0	0	0	0
Oceanobacillus	0	0	0	0	0,000178795	0	0	0	0	0	0	0	4,47E-05	0	0
Sphingopyxis	0	0	0	0	0	8,94E-05	0	0	0	0	0,000134096	0	0	0	0
Pseudoxanthomonas	0	0	0	0	0	0	0	8,94E-05	0	0	0,000134096	0	0	0	0
Treponema_2	0	0	4,47E-05	0	0	0	0	0	0	0	0	0	0,000178795	0	0
Actinomycetospora	0	4,47E-05	0	0	0	0	0	0	0	0	0,000178795	0	0	0	0
Jatrophihabitans	0	0	0,000223494	0	0	0	0	0	0	0	0	0	0	0	0
Kineococcus	0	0	0	0	0	0	0	0,000223494	0	0	0	0	0	0	0
Spirochaeta_2	0	0	0,000223494	0	0	0	0	0	0	0	0	0	0	0	0
Candidatus_Alysiosphaera	0	0	0	0	0	0	0	0	0	0	0,000223494	0	0	0	0
Dokdonella	0	0	0,000223494	0	0	0	0	0	0	0	0	0	0	0	0
Lachnospiraceae_NC2004_group	0	0	0	0,000223494	0	0	0	0	0	0	0	0	0	0	0
uncultured_Acidobacteria_bacterium	0	0	0	0	0	0	0	0	0	0	0,000223494	0	0	0	0
Paracoccus	0	0	0	0	0,000223494	0	0	0	0	0	0	0	0	0	0
Aetherobacter	0	0	0	0	0	0	0	0	0	0	0,000223494	0	0	0	0
mle1-7	0	0	0,000223494	0	0	0	0	0	0	0	0	0	0	0	0
Aerococcus	0	4,47E-05	8,94E-05	0	0	0	0	0	0	0	0	4,47E-05	0	0	0
Lysinibacillus	0	0	0	0	0	0	4,47E-05	0	8,94E-05	4,47E-05	0	0	0	0	0
[Eubacterium]_fissicatena_group	0	0	0	4,47E-05	0	0	0	0	0	8,94E-05	0	4,47E-05	0	0	0
Geodermatophilus	0	0	0	0	0,000134096	0	0	0	4,47E-05	0	0	0	0	0	0
Kibdelosporangium	0	0	0	0,000134096	0	0	0	0	4,47E-05	0	0	0	0	0	0
Sphingoaurantiacus	0	0	0	0	0	0	0	0,000134096	0	0	0	0	0	4,47E-05	0
OM27_clade	0	0	0	0	0	0	0	0	0	0	0,000178795	0	0	0	0
Edaphobacter	0	0	0	0	0	0	0	0	0	0	0,000178795	0	0	0	0
Fermentimonas	0	0	0,000178795	0	0	0	0	0	0	0	0	0	0	0	0
Olivibacter	0	0	0	0	0	0	0	0	0	0	0	0,000178795	0	0	0
Geobacter	0	0	0	0	0	0	0	0	0	0	0	0	0,000178795	0	0
Luteimonas	0	0	0	0	0	0	0	0	0	0	0	0,000178795	0	0	0
Prevotella	0	0	0	0	0	0	0	0	0	0	0	0	0,000178795	0	0
Ilumatobacter	0	0	0	0	0	0	0	0	0	0	0,000178795	0	0	0	0
[Mycoplasma]_group	0	0	0,000178795	0	0	0	0	0	0	0	0	0	0	0	0
Ruegeria	0	0	0	0	0	0	0	0	0	0	0,000178795	0	0	0	0
Thermobispora	4,47E-05	0	0	0	0	0	0	0	0	0	0	0	4,47E-05	4,47E-05	0
Ralstonia	0	0	4,47E-05	0	0	4,47E-05	4,47E-05	0	0	0	0	0	0	0	0
Nitrospira	0	0	0	0	0	0	4,47E-05	0	0	0	4,47E-05	0	4,47E-05	0	0
Xanthomonas	0	0	0	0	0	4,47E-05	0	0	0	0	0	8,94E-05	0	0	0
Lachnoclostridium_10	0	4,47E-05	0	0	0	0	0	0	0	0	0	0	8,94E-05	0	0
Fournierella	0	0	0	8,94E-05	0	0	0	0	0	0	0	0	0	4,47E-05	0
Pseudochelatococcus	0	0	8,94E-05	0	0	0	0	0	0	0	4,47E-05	0	0	0	0
Granulicatella	0	8,94E-05	0	4,47E-05	0	0	0	0	0	0	0	0	0	0	0
Empedobacter	0	0	0	0	0	0	0	0	0	0	0	8,94E-05	4,47E-05	0	0
GWE2-42-42	0	0	0,000134096	0	0	0	0	0	0	0	0	0	0	0	0
Dinghuibacter	0	0	0	0	0	0	0	0	0	0	0,000134096	0	0	0	0
uncultured_forest_soil_bacterium	0	0	0,000134096	0	0	0	0	0	0	0	0	0	0	0	0
Thermomonospora	0	0	0	0	0	0	0	0	0	0	0	0	0,000134096	0	0
Oceanimonas	0	0	0	0	0	0	0	0	0	0	0	0,000134096	0	0	0
Thauera	0	0	0	0	0	0	0	0	0	0	0,000134096	0	0	0	0
uncultured_Acidimicrobiales_bacterium	0	0	0	0	0	0	0	0	0	0	0,000134096	0	0	0	0
Taonella	0	0	0	0	0	0	0	0	0	0	0,000134096	0	0	0	0
Saccharopolyspora	0	0	0,000134096	0	0	0	0	0	0	0	0	0	0	0	0
Lechevalieria	0	0	0	0	0	0	0	0	0	0	0,000134096	0	0	0	0
Erysipelothrix	0	0	0	0	0	0	0	0	0	0	0	0	0	0	0,000134096
Family_XIII_UCG-001	0	0	0	0	0	0	0,000134096	0	0	0	0	0	0	0	0
Marmoricola	0	0	0,000134096	0	0	0	0	0	0	0	0	0	0	0	0
Moheibacter	0	0	0	0	0	0	0	0	0	0	0	4,47E-05	4,47E-05	0	0
Campylobacter	0	0	0	4,47E-05	0	0	0	0	0	4,47E-05	0	0	0	0	0
Rahnella	0	0	0	0	0	0	4,47E-05	0	0	0	0	4,47E-05	0	0	0
Corynebacterium	0	0	4,47E-05	4,47E-05	0	0	0	0	0	0	0	0	0	0	0
Prevotellaceae_Ga6A1_group	0	0	0	4,47E-05	0	0	4,47E-05	0	0	0	0	0	0	0	0
uncultured_compost_bacterium	0	0	0	0	0	0	0	0	0	0	0	0	8,94E-05	0	0
Dactylosporangium	0	0	0	0	8,94E-05	0	0	0	0	0	0	0	0	0	0
Actinoallomurus	0	0	0	0	0	0	0	0	0	0	8,94E-05	0	0	0	0
Acidaminococcus	0	0	0	0	0	0	8,94E-05	0	0	0	0	0	0	0	0
Candidatus_Entotheonella	8,94E-05	0	0	0	0	0	0	0	0	0	0	0	0	0	0
Actinomadura	0	0	0	0	0	0	0	0	0	0	8,94E-05	0	0	0	0
Ferruginibacter	0	0	0	0	0	0	0	0	0	0	8,94E-05	0	0	0	0
Desulfomonile	0	0	8,94E-05	0	0	0	0	0	0	0	0	0	0	0	0
Solobacterium	0	0	0	0	0	0	0	0	0	8,94E-05	0	0	0	0	0
Plastorhodobacter	0	0	0	0	0	0	8,94E-05	0	0	0	0	0	0	0	0
Pseudoflavitalea	0	0	0	8,94E-05	0	0	0	0	0	0	0	0	0	0	0
Pelagibacterium	0	0	0	0	0	0	0	0	0	0	0	0	8,94E-05	0	0
Oscillospira	0	0	0	8,94E-05	0	0	0	0	0	0	0	0	0	0	0
Legionella	0	0	0	0	0	0	0	0	0	0	8,94E-05	0	0	0	0
Mariniflexile	8,94E-05	0	0	0	0	0	0	0	0	0	0	0	0	0	0
Sporacetigenium	0	0	0	0	0	0	0	0	0	0	8,94E-05	0	0	0	0
Porphyromonas	0	0	8,94E-05	0	0	0	0	0	0	0	0	0	0	0	0
Syntrophus	0	0	0	0	0	0	4,47E-05	0	0	0	0	0	0	0	0
Albirhodobacter	0	0	0	0	0	0	0	0	0	0	0	4,47E-05	0	0	0
Oribacterium	0	0	0	4,47E-05	0	0	0	0	0	0	0	0	0	0	0
Ornithinibacillus	0	0	0	0	0	0	0	0	0	0	0	4,47E-05	0	0	0
Ornithinimicrobium	0	0	0	0	0	0	0	0	0	0	4,47E-05	0	0	0	0
uncultured_actinobacterium	0	0	0	0	0	0	0	0	0	0	4,47E-05	0	0	0	0
Erythrobacter	0	0	0	0	0	0	0	4,47E-05	0	0	0	0	0	0	0
Thermobifida	0	0	0	0	0	0	0	0	0	4,47E-05	0	0	0	0	0
Rhodoferax	0	0	0	0	0	0	0	0	0	0	4,47E-05	0	0	0	0
Peptostreptococcus	0	0	0	4,47E-05	0	0	0	0	0	0	0	0	0	0	0
Phaeodactylibacter	0	0	0	0	0	0	0	0	0	0	4,47E-05	0	0	0	0
Tissierella	0	0	0	0	0	0	0	0	0	0	0	4,47E-05	0	0	0
uncultured_proteobacterium	0	0	0	0	0	0	0	0	4,47E-05	0	0	0	0	0	0
Achromobacter	0	0	4,47E-05	0	0	0	0	0	0	0	0	0	0	0	0
Allobaculum	0	4,47E-05	0	0	0	0	0	0	0	0	0	0	0	0	0
Amaricoccus	0	0	0	0	0	0	0	0	0	0	4,47E-05	0	0	0	0
Jeotgalicoccus	0	0	0	0	0	0	0	0	0	0	4,47E-05	0	0	0	0
Oligoflexus	0	0	0	0	0	0	0	0	0	0	0	4,47E-05	0	0	0
Niastella	0	0	0	0	0	0	0	0	0	0	4,47E-05	0	0	0	0
Modestobacter	0	0	4,47E-05	0	0	0	0	0	0	0	0	0	0	0	0
Duganella	0	0	0	0	0	0	0	0	0	0	0	4,47E-05	0	0	0
Abiotrophia	0	0	0	4,47E-05	0	0	0	0	0	0	0	0	0	0	0
Macrococcus	0	0	0	0	0	0	0	0	0	4,47E-05	0	0	0	0	0
Longispora	0	0	0	0	0	0	0	0	0	0	4,47E-05	0	0	0	0
Bdellovibrio	0	0	0	4,47E-05	0	0	0	0	0	0	0	0	0	0	0
Bergeyella	0	0	0	0	0	0	0	0	0	0	4,47E-05	0	0	0	0
Ruminococcaceae_UCG-008	0	0	0	4,47E-05	0	0	0	0	0	0	0	0	0	0	0
Butyrivibrio	0	0	0	0	0	0	0	0	0	0	4,47E-05	0	0	0	0
Lachnospiraceae_UCG-009	0	0	0	0	0	0	0	0	0	4,47E-05	0	0	0	0	0
[Eubacterium]_nodatum_group	0	0	0	4,47E-05	0	0	0	0	0	0	0	0	0	0	0
Kocuria	0	0	0	0	0	0	4,47E-05	0	0	0	0	0	0	0	0
Microvirga	0	0	0	0	0	0	0	0	0	0	4,47E-05	0	0	0	0
