# Supplementary figures and images for: Influence of aerobic exercise training on mice gut microbiota in Parkinson’s disease
Source: Turk J Biol. 2022 Apr 25;46(4):288–97. doi: 10.55730/1300-0152.2617 (PMC10388120; doi:10.55730/1300-0152.2617)

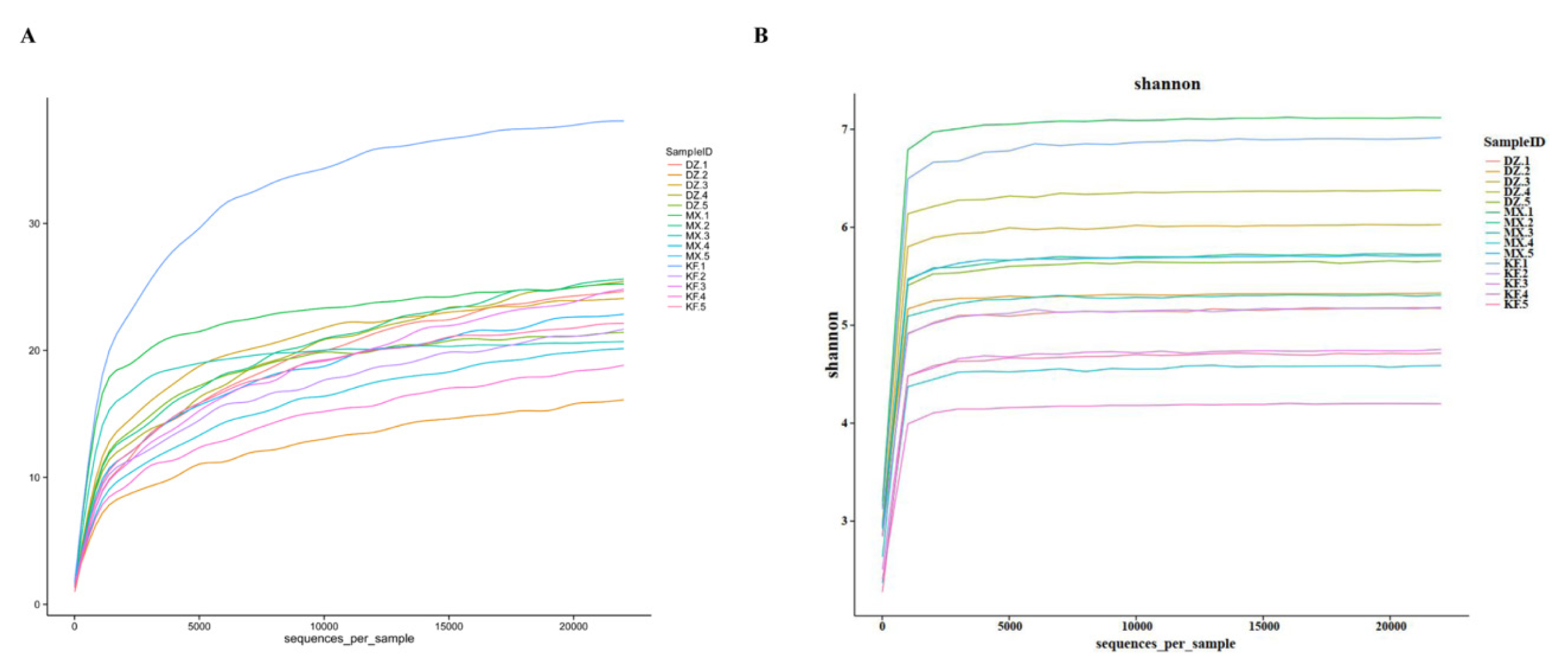

Supplement: Figure S1 — The validation of 16S rRNA sequencing data analysis. (A) Diversity index rarefaction curve assessing the number sequences. (B) Rarefaction curve for Shannon index evaluating the relative bacterial richness of the number sequences. [file turkjbiol-46-4-288s1.tif]

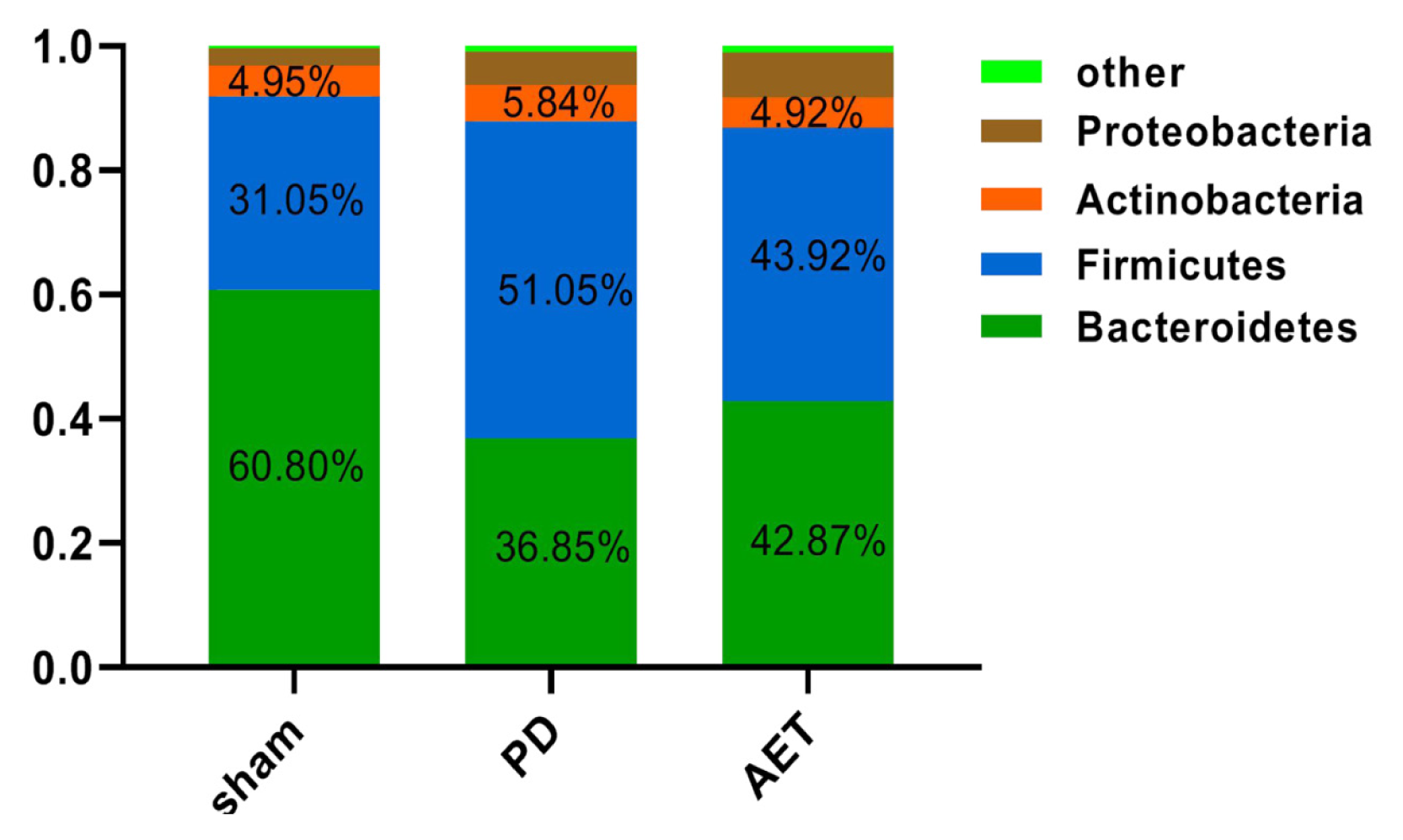

Supplement: Figure S2 — Composition proportions of fecal microbiota of the phyla in sham-operated mice, PD mice, mice receiving AET. [file turkjbiol-46-4-288s2.tif]

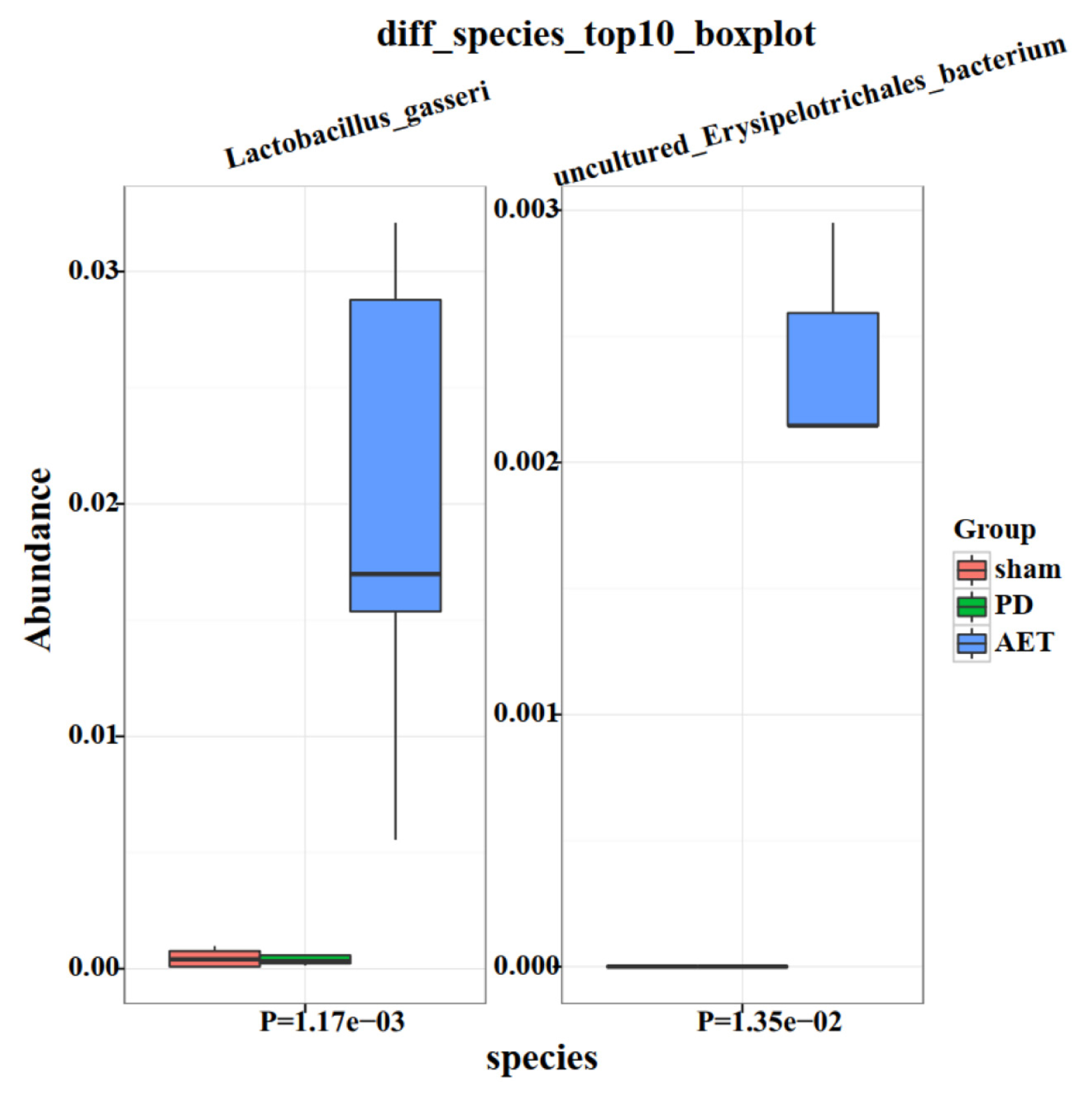

Supplement: Figure S3 — Abundance of top 10 species in sham, PD and AET groups. [file turkjbiol-46-4-288s3.tif]
